# Characterizing noise structure in single-cell RNA-seq distinguishes genuine from technical stochastic allelic expression

**DOI:** 10.1038/ncomms9687

**Published:** 2015-10-22

**Authors:** Jong Kyoung Kim, Aleksandra A. Kolodziejczyk, Tomislav Illicic, Sarah A. Teichmann, John C. Marioni

**Affiliations:** 1 European Molecular Biology Laboratory-European Bioinformatics Institute (EMBL-EBI), Wellcome Trust Genome Campus, Cambridge CB10 1SD, UK; 2 Wellcome Trust Sanger Institute, Wellcome Trust Genome Campus, Cambridge CB10 1SA, UK

## Abstract

Single-cell RNA-sequencing (scRNA-seq) facilitates identification of new cell types and gene regulatory networks as well as dissection of the kinetics of gene expression and patterns of allele-specific expression. However, to facilitate such analyses, separating biological variability from the high level of technical noise that affects scRNA-seq protocols is vital. Here we describe and validate a generative statistical model that accurately quantifies technical noise with the help of external RNA spike-ins. Applying our approach to investigate stochastic allele-specific expression in individual cells, we demonstrate that a large fraction of stochastic allele-specific expression can be explained by technical noise, especially for lowly and moderately expressed genes: we predict that only 17.8% of stochastic allele-specific expression patterns are attributable to biological noise with the remainder due to technical noise.

## Abstract (web summary)

Single-cell RNA-sequencing (scRNA-seq) can be applied to dissect the kinetics of gene expression and patterns of allele-specific expression. Here, Kim *et al.* report a generative statistical model that can separate biological variability from technical noise by quantifying technical noise using external RNA spike-ins.

## Graphical abstract

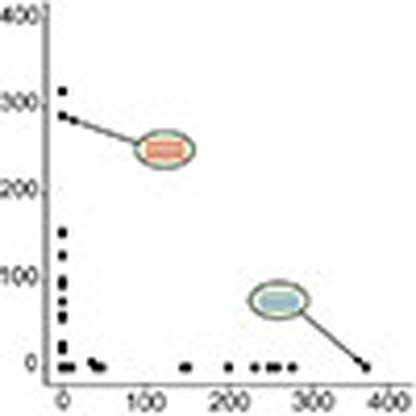

Profiling the transcriptomes of individual cells via single-cell RNA-sequencing (scRNA-seq) allows the functional role of heterogeneity in gene expression levels between cells to be investigated in early development, in cancer and during tissue differentiation^1^,^2^,^3^,^4^,^5^,^6^,^7^,^8^,^9^. Current scRNA-seq protocols require amplification of the minute amount of mRNA present in an individual cell so that next-generation sequencing libraries can be prepared. More specifically, following cell lysis and reverse transcription of the poly-adenylated fraction of RNA molecules, PCR or *in vitro* transcription is used to amplify cDNA molecules. In combination, these steps contribute to substantial increases in the level of technical noise relative to bulk-level RNA-seq^5^,^10^,^11^,^12^.

Several strategies have been proposed to reduce or eliminate technical noise in scRNA-seq protocols. First, a large fraction of polyadenylated RNA is stochastically lost during sample preparation steps including cell lysis, reverse transcription and amplification^5^. Studies where sample preparation was performed in microlitre volumes and cells are hand-picked reported a capture efficiency on the order of 10% (refs 5, 10). In contrast, nanolitre-volume scRNA-seq using microfluidic platforms that automate sample preparation showed an improved capture efficiency of up to 40% (ref. 11), substantially reducing the bias introduced by stochastic RNA loss. Second, the linear or exponential amplification, in conjunction with the stochastic RNA loss, introduces amplification bias, especially for lowly expressed genes^12^. A recent approach that counts the absolute number of molecules per gene using unique molecular identifiers facilitated modelling of the amplification bias and reduced the overall levels of technical noise^10^,^13^. Finally, scRNA-seq protocols that profile full-length transcripts suffer from a 3′-end bias due mainly to inefficiencies in reverse transcription and incomplete RNA degradation^14^, although recent developments have led to improvements^15^,^16^. Despite this limitation, full-length protocols are popular as they allow transcript isoform identification^3^ and measurement of allele-specific expression (ASE) by using single-nucleotide polymorphisms (SNPs)^17^ in the coding sequence. However, owing to challenges in processing the small quantity of starting molecules, current scRNA-seq protocols have substantially increased levels of technical noise relative to bulk RNA-seq. Consequently, accurately quantifying the contributions of technical and biological noise to variability in gene expression levels across cells at both the whole gene and the allele-specific level is challenging.

To date, computational strategies have focused on using extrinsically spiked-in molecules to model background (technical) noise in scRNA-seq data^10^,^12^. However, current approaches either fail to account for the substantial differences in technical noise between cells^12^ or make strong parametric assumptions about the relationship between variation and gene expression^10^. In the context of ASE at the single-cell level, this is an extremely important problem, as failing to correctly account for such features might lead to the incorrect identification of stochastic ASE. Indeed, to date, there has been no formal attempt to incorporate measurements of technical noise from extrinsic spike-in molecules into the identification of stochastic ASE.

To address this problem, we develop a generative model, which extends and integrates key aspects of previous analytical approaches (Supplementary Notes 1–3). Our approach is based upon an explicit probabilistic model, which allows scRNA-seq data to be simulated under a variety of assumptions and then contrasted to true data. To validate our approach, we distinguish biological from technical variability in scRNA-seq data generated from mouse embryonic stem cells (mESCs) cultured in serum/leukaemia inhibitory factor (LIF) or 2i/LIF conditions^10^. Using single-molecule fluorescent *in situ* hybridization (smFISH) data, we demonstrate that our approach better estimates biological variability than previously described computational strategies, especially for lowly expressed genes. Having validated our model, we use it to explore the influence of technical and biological noise upon measurements of ASE in mESCs derived from a first-generation cross of two inbred mouse strains. Our analysis reveals that a substantial degree of apparent stochastic ASE can be explained by technical noise, with important implications for studying ASE in single cells.

## Results

### Overview of the method

We developed a statistical method to quantify biological noise by decomposing the total variance of each gene’s expression across cells into biological and technical components, while minimizing assumptions on the form of distributions for each noise component.

**Figure 1:**
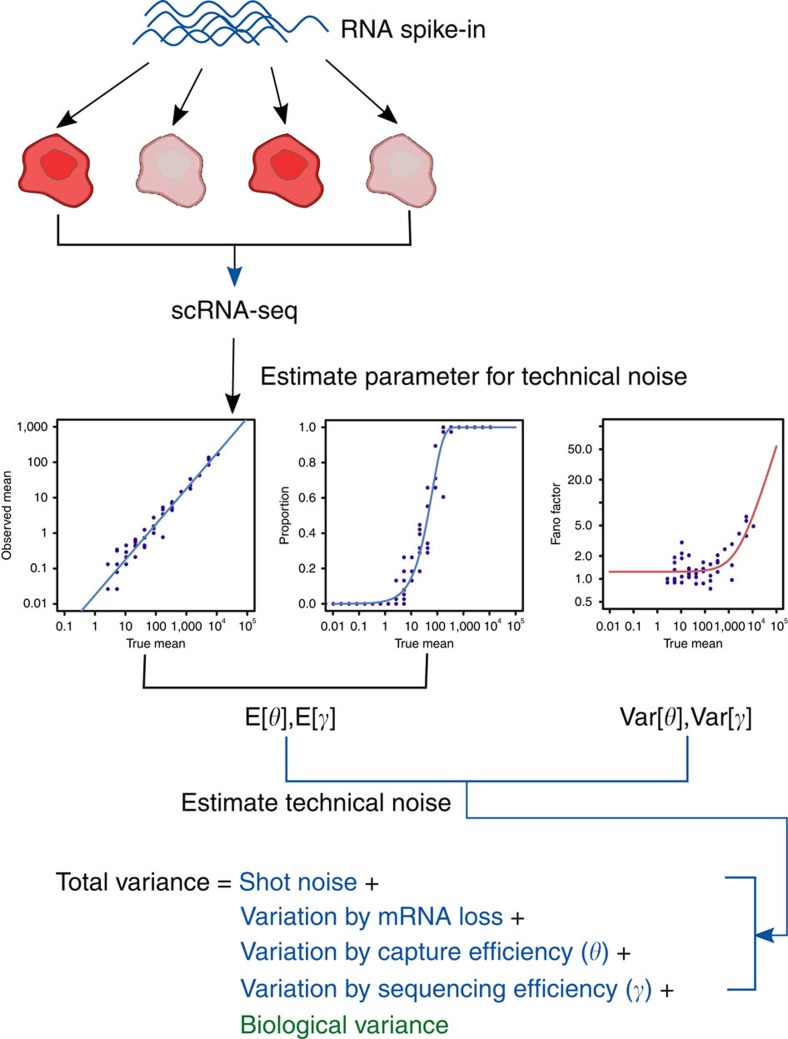
Schematic representation of the noise decomposition method. With the help of external RNA spike-in molecules, added at the same quantity to each cell’s lysate, we first estimate four parameters capturing technical variability, which are the expectation and variance of capture (*θ*) and sequencing (*γ*) efficiency. Then, by the general variance decomposition formula, the total observed variance of read counts can be decomposed into technical (blue) and biological (green) variance terms. The estimate of biological variance can be obtained by subtracting technical variance terms from the total observed variance. Shot noise (or Poisson noise) is cell-to-cell variability that can be modelled by a Poisson process.

Our method uses external RNA spike-in molecules, added at the same quantity to each cell’s lysate, to model the expected technical noise across the dynamic range of gene expression. It assumes a probabilistic model that represents the underlying generative process of observed counts in scRNA-seq data (Supplementary Notes 1, 2 and Supplementary Fig. 1). The generative model captures two major sources of technical noise: (i) stochastic dropout of transcripts during the sample preparation procedure and (ii) shot noise. Critically, to model cell-to-cell differences in capture efficiency and shot noise, we allow these quantities to vary between cells. It should be noted that the model cannot capture stochastic RNA losses that arise because of inefficient cell lysis as the external RNA spike-ins are added to the lysis buffer. Third, our model decomposes the total variance into multiple terms that correspond to different sources of variation^18^, where all of the necessary parameters can be estimated using the external RNA spike-ins (Supplementary Note 3 and Fig. 1). The biological variance can be estimated by subtracting variance terms corresponding to technical noise from the total observed variance. Finally, our model allows scRNA-seq data to be simulated under various assumptions, which can be applied to distinguish genuine stochastic ASE across cells from artefacts induced by technical noise.

### Estimating biological noise

We analysed two publicly available scRNA-seq data sets where individual mESCs cultured in two different conditions (serum/LIF or 2i/LIF) were profiled using a unique molecular identifier protocol^10^. The libraries were prepared in two batches, the first containing 40 mESCs cultured in 2i and 40 mESCs cultured in serum and the second containing the remaining cells. Each batch was split into two and sequenced on different lanes of an Illumina HiSeq2500 (ref. 10).

To ensure the quality of the data, following the approach of Grün *et al.*^10^, we discarded cells with fewer than 500 sequenced transcripts for External RNA Control Consortium (ERCC) spike-ins and 10,000 sequenced transcripts for endogenous genes. In the first batch, 38 2i and 37 serum mESCs remained after filtering with 38 2i and 18 serum cells remaining in the second batch. We did not apply an additional filter used by Grün *et al.*^10^, who discarded cells with fewer than 10 sequenced transcripts for *Pou5f1* in 2i and 25 in serum. We did this to avoid the possibility of a genuine subpopulation of cells being overlooked.

As the cells were prepared in multiple independent batches, we used the expression levels of the ERCC transcripts to explore whether technical batch effects were confounded with the biological signal of interest (Supplementary Note 4). Although all cells were spiked with the same volume of the ERCC spike-in mix, we observed that cells cluster by batch first and only subsequently by culture condition, indicating the presence of strong technical effects (Supplementary Fig. 2a). To identify the source of this variation, we estimated the strength of the linear relationship between the observed and expected spike-in counts (which incorporates all sources of technical variability except for cell lysis inefficiencies), denoted by *E[η]* (Supplementary Note 1 and 2), separately for each batch and observed considerable variation (*E[η]* ranged from 0.0177 to 0.0361 indicating substantial differences in both capture and sequencing efficiency between batches). We speculated that the variability in *E[η]* was the main source of batch effects. Indeed, when we normalized the raw number of sequenced ERCC transcripts by dividing by *E[η]* (separately for each batch), the batch effects were removed (Supplementary Fig. 2b). Hence, we used the normalized number of sequenced transcripts in all downstream analyses.

To validate our approach for estimating technical noise, we compared the expected technical noise derived from the generative model with the observed noise from the ERCC spike-ins. Our model captured the mean-variance relationship for estimating technical noise successfully across the whole dynamic range of expression levels (Supplementary Figs 3–6 and Supplementary Note 5). For each gene, we used our model to estimate the contribution of technical and biological variability to the overall variability in its expression across cells (Supplementary Figs 5 and 6).

**Figure 2:**
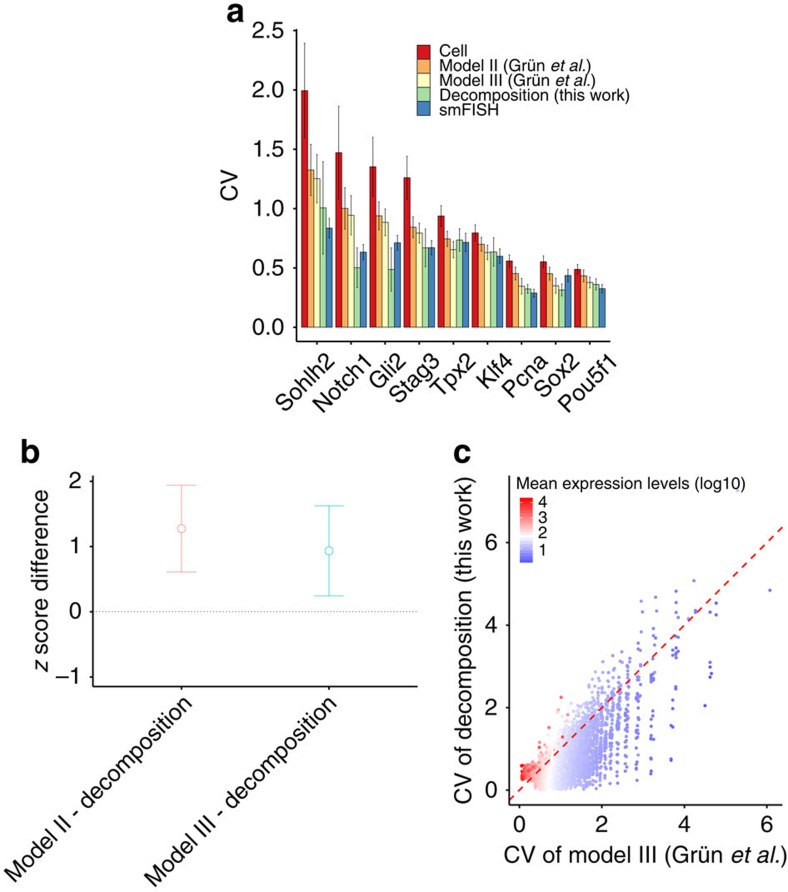
Validation of estimated biological noise of 2i-grown mESCs by single-molecule FISH. (**a**) Bar plot depicts the measured coefficient of variation (CV) (*y* axis) of chosen genes by each method: total noise by scRNA-seq (Cell); Models II and III of Grün *et al.*^10^; our noise decomposition method (Decomposition); single cell FISH (smFISH). Genes chosen by Grün *et al.*^10^ to cover a dynamic range of gene expression are sorted by their expression levels: lowly expressed genes (*Sohlh2*, *Notch1*, *Gli2* and *Stag3*), moderately expressed genes (*Tpx2*) and highly expressed genes (*Pou5f1*, *Sox2*, *Pcna2* and *Klf4*). *Notch1* is not available in serum-grown mESCs of Grün *et al.*^10^ Error bars represent standard deviation (s.d.): bootstrap s.d. for our predictions; s.d. derived from estimated standard errors of the parameters of a negative binomial distribution for other methods. (**b**) Comparison of models for the deviation of the model estimates of CV from the smFISH estimates of CV using z-scores of lowly expressed gene. To compare the accuracy of the model estimates of CV, we performed a one-tailed paired *t*-test between two paired sets (corresponding to different methods) of z-scores of genes for each group. Here the *z*-score is defined as |*x*_*i*_-*μ*_*i*_|/*σ*_*i*_, where *x*_i_ is the model estimate of CV of gene *i*, *μ*_*i*_ is the smFISH estimate of CV of gene *i* and *σ*_*i*_ is the standard deviation of the model estimate of CV of gene *i*. As the lower *z*-score means more accurate estimate of biological CV in terms of smFISH measurements, we set the alternative hypothesis to state that the *z*-scores of our method is less than that of other methods. For lowly expressed genes, our method outperforms the deconvolution-based methods (*P*=0.0166 between model II and ours, *P*=0.0385 between model III and ours). Error bars represent 95% confidence intervals. (**c**) Comparison of biological estimates of CV between model III of Grün *et al.*^10^ and our noise decomposition method.

We assessed our model’s performance by comparing its output with gold standard estimates of biological variability generated for nine genes using smFISH (Fig. 2 for 2i-grown mESCs and Supplementary Fig. 7 for serum-grown mESCs). To compare our biological noise predictions with a recently proposed method^10^, we used the same set of cells (74 for 2i and 44 for serum) by applying the additional filter based on the number of sequenced transcripts of *Pou5f1*. In general, we observed excellent concordance between our estimates of biological noise and those derived from smFISH. For lowly expressed genes (*Sohlh2*, *Notch1*, *Gli2* and *Stag3*), our method outperforms the deconvolution-based methods (*P*<0.05 by one-tailed paired *t*-test; Fig. 2a,b). However, for the more highly expressed genes for which smFISH data are available, there is no significant difference between methods at a significance level of 0.1. Importantly, unlike the previous state-of-the-art approach^10^, we did not systematically overestimate biological noise for lowly expressed genes (*Sohlh2*, *Notch1*, *Gli2* and *Stag3*), primarily because we did not assume transcript counts are the sum of two independent counts capturing technical and biological processes that were modelled by a negative binomial distribution (Fig. 2c). More generally, for lowly expressed genes (<20th percentile), 11.9% of variance in their expression across cells can be attributed to biological variability on average, as opposed to 55.4% for highly expressed genes (>80th percentile).

### Stochastic ASE

Single-cell measurements of ASE provide a valuable resource for studying transcriptional diversity^17^. One interesting pattern is monoallelic expression, where only one allele of a gene is expressed. The choice of which allele to actively transcribe can be determined by the parental origin of each allele (for example, genomic imprinting) or randomly (for example, X chromosome inactivation). Random monoallelic expression has also been reported at some autosomal genes where it is established stochastically during differentiation after which it is maintained through subsequent mitotic cell divisions^19^. Interestingly, a recent study used scRNA-seq data to show that many autosomal genes (12–24%) display transient monoallelic expression in individual cells of mouse preimplantation embryos, where the stochastic RNA loss was modelled by splitting each cell’s lysate into two fractions of equal volume^17^.

**Figure 3:**
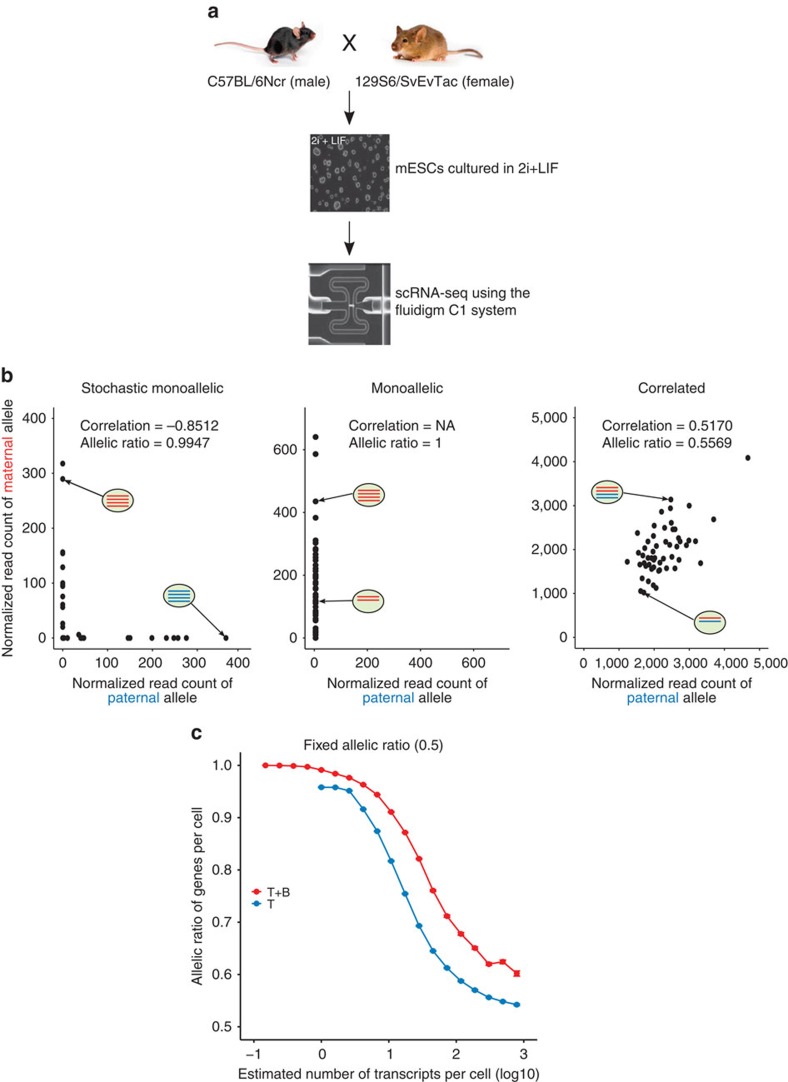
Stochastic monoallelic expression. (**a**) Experimental design of allele-specific scRNA-seq. (**b**) Examples of genes showing stochastic monoallelic, monoallelic and correlated allelic expression. In each scatter plot, black dots represent normalized read counts of maternal (red in an oval) and paternal (blue in an oval) alleles of single cells (depicted as an oval) of a single gene. In contrast to monoallelic expression (the most expressed allele is the same across cells), the most expressed allele in stochastic monoallelic expression is different across cells. (**c**) Mean fraction of most expressed alleles for SNPs binned by expression levels assuming each allele is expressed at the same level, simulated from the ‘T’ model (solid blue line) or the ‘T+B’ model (solid red line). Error bars denote 95% confidence intervals by bootstrap (100 bootstrap samples). Mouse images reproduced with permission from the Jackson Laboratory.

In bulk-based studies, ASE describes differences in the mean level of expression between the two alleles. scRNA-seq allows a more nuanced understanding: we define stochastic ASE as excessive variability in the ratio of the expression level of the paternal (or maternal) allele between cells after controlling for mean allelic expression levels (Fig. 3b). To date, heuristic approaches based on the fraction of reads mapped to each allele across cells have been used to identify genes displaying stochastic ASE. Even though these approaches usually filter out lowly expressed genes, this has not allowed technical noise, a potentially major contributor to perceived stochastic ASE, to be properly accounted for.

To explore this, we considered newly generated scRNA-seq data from 54 mouse ESCs cultured in 2i and derived from a first-generation intercross between two inbred strains (C57BL/6Ncr male × 129S6/SvEvTac female, Fig. 3a). We initially sequenced the cDNA from cells captured using a 96-cell Fluidigm C1 IFC and filtered out low-quality cells, resulting in 54 cells that were used in downstream analysis. The 92 ERCC spike-in molecules were used to quantify the extent of technical noise (Methods). In total, 28,912 transcripts contained at least one SNP that could be used to distinguish between the strains, meaning that ASE could be quantified for 7,385 (29.6%) of 24,941 expressed genes.

We applied the model introduced earlier by first estimating, for each gene, the mean expression level and biological variance of the maternal and paternal alleles before simulating 54 pseudo cells assuming only technical variability (‘T’) or assuming both technical and biological variability (‘T+B’; Supplementary Fig. 8). We simulated the single-cell data using the generative process of our model under the assumption that the two alleles of a gene are expressed independently (Supplementary Note 6 and Supplementary Fig. 9) and showed that the simulated data reflect the real scRNA-seq data at the level of both mean expression and variability (Supplementary Fig. 10).

To examine the effect of technical noise on the allelic ratio (defined as the expression of the most expressed allele divided by the total across both alleles), we simulated single-cell data under the ‘T’ model where each allele was expressed at the same level (Supplementary Note 6). Lowly expressed genes frequently displayed stochastic monoallelic expression (90–100% observed allelic ratio, Fig. 3c), where lowly expressed genes are defined as those with an estimated number of transcripts per cell <1. Importantly, total noise was dominated by technical noise (40–100%) for lowly expressed genes (Supplementary Fig. 11a), indicating that ASE observed in this range of expression values is unlikely to be genuine. Although the observed allelic ratios of genes successively decreased towards the true value as the expression levels increased, the observed allelic ratio did not converge to the true value even at the maximum number of transcripts per cell (∼1,000, Fig. 3c), potentially suggesting the difficulty of accurately estimating the true allelic ratios of genes with the balanced expression of both alleles because of the technical variability present in scRNA-seq data.

We also examined the combined effect of technical and biological noise on the estimated allelic ratio by simulating single-cell data under the ‘T+B’ model (Supplementary Note 6), again assuming that each allele was expressed at the same level. Including biological noise yielded allelic ratios that are larger than those observed in the ‘T’ model and are above the true value across the whole dynamic range of expression levels, suggesting that we cannot ignore biological noise even at the maximum expression level (Fig. 3c). More genes with an estimated number of transcripts per cell <10 showed monoallelic expression (90–100% observed allelic ratio) compared with the ‘T’ model.

### Identifying genuine stochastic ASE

We observed that 72.7% of all expressed SNPs in 54 cells had an allelic ratio above 0.95, suggesting that 99.7% of expressed genes display monoallelic expression in at least one cell (this percentage falls to 99.3% when only the most expressed SNP per gene is considered). Previous reports obtained similar results in other cell types and suggested that the majority of this ASE is stochastic^20^.

Of 75,872 expressed SNPs, 99.4% had an allelic ratio larger than 0.95 in at least one cell (Supplementary Fig. 11d). When we simulated single-cell data under the ‘T’ model with the allelic ratio fixed to 50%, we found 47.2% had an allelic ratio above 0.95 in at least one cell, indicating that about half of SNPs displaying monoallelic expression in cells are driven by technical noise. When we allowed biological variability and fixed the allelic ratio at 50% (‘T+B’ in Supplementary Fig. 11d), 69.1% showed monoallelic expression in at least one cell, suggesting that biological variability accounts for about 20% of the expressed SNPs displaying monoallelic expression in cells. The remaining 30% could be potentially explained by differences in the mean expression levels of the two alleles.

We formalized the above arguments within the null hypothesis significance testing framework as follows. To identify genes showing stochastic ASE not explained by technical noise and differences in the mean expression levels between the maternal and paternal alleles, we separately estimated the mean expression of the two alleles before simulating 54 pseudo cells assuming only technical variability (Supplementary Notes 6,7 and Supplementary Fig. 8). For each pseudo cell, we then calculated the allelic ratio for all simulated genes and computed its mean value across the simulated cells. We repeated this process 10,000 times, yielding a null distribution of simulated ratios, which we contrasted with the observed ratio. Notice that we accounted for differences in the mean level of expression between the two alleles by separately estimating the mean expression of both alleles (instead of fixing the allelic ratio at 50%) since we defined stochastic ASE as excessive variability in differences in the expression levels between the two alleles across single cells, after controlling for differences in average allelic expression levels.

**Figure 4:**
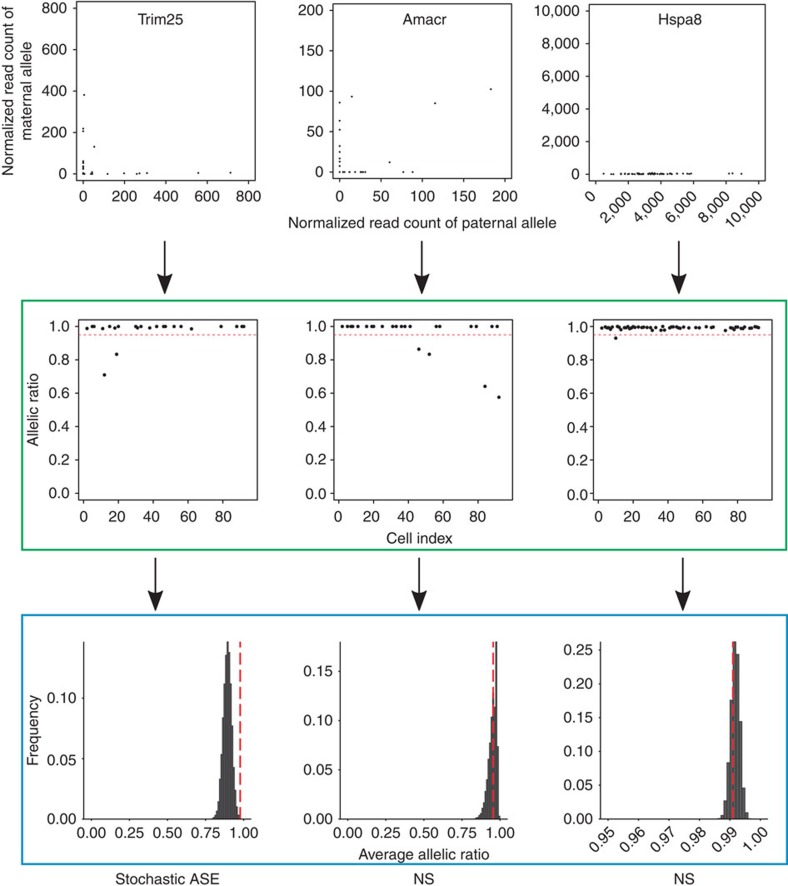
Testing stochastic allelic-specific expression. In the upper panel, each dot in the three scatter plots (for SNPs assigned to *Trim25*, *Amacr*, or *Hspa8*) represents the expression value of a single cell, where the *x* axis represents the normalized read count of the paternal allele and the *y* axis represents the normalized read count of the maternal allele of a single gene. In the middle panel highlighted by the green box, each scatter plot shows the allelic ratios (*y* axis) of cells (*x* axis, cell index from 1 to 96) of a single gene. The horizontal dashed red line represents the cutoff of 0.95 to define monoallelic expression. All three genes display monoallelic expression in at least one cell by this criterion. In the lower panel highlighted by the blue box, we simulated 54 pseudo cells assuming only technical noise and calculated the average allelic ratio across the pseudo cells. We repeated this process 10,000 times and computed the empirical *P*-value of the observed average allelic ratio (vertical dashed red line) based on a null distribution of simulated ratios (black). Of the three genes, only *Trim25* has a higher average allelic ratio than expected by chance, suggesting it displays stochastic allele-specific expression. NS, not significant.

As an example, *Trim25* shows a strong ‘L’-shaped pattern between the two alleles, and most individual cells have an allelic ratio above 0.95. It also has a higher average allelic ratio than expected by chance, suggesting it displays stochastic ASE (in the left column of Fig. 4). However, even though *Amacr* shows a similar expression pattern to *Trim25*, its average allelic ratio is not significantly higher than would be expected by chance, indicating that the monoallelic expression of *Amacr* in cells is driven by technical variability (in the middle column of Fig. 4). Notice that we do not consider monoallelic expression observed in bulk-based studies (for example, genomic imprinting) as stochastic since differences in the expression levels between the two alleles are conserved across cells (*Hspa8* in the right column of Fig. 4).

**Figure 5:**
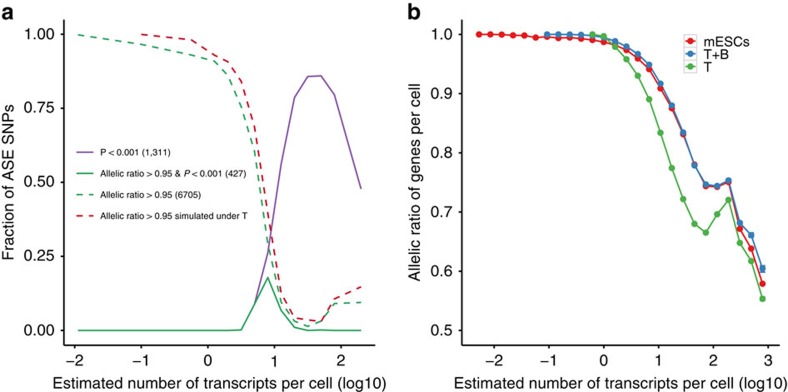
Distinguishing genuine from technical allelic expression patterns. (**a**) Mean fraction of SNPs showing stochastic ASE as a function of overall gene expression. Colours indicate different approaches for calling stochastic ASEs. Numbers in parentheses represent the number of genes identified by each approach. (**b**) Mean fraction of most expressed alleles for SNPs binned by expression levels. The mean expression levels of both alleles were separately estimated. Colours indicate different approaches for computing the mean fraction by scRNA-seq measurements (mESCs), ‘T’ model and ‘T+B’ model. Error bars denote 95% confidence intervals by bootstrap (100 bootstrap samples).

Of 7,385 genes with one or more expressed SNPs (Supplementary Fig. 12), 1,311 (17.8%) showed more variation across cells than expected by chance (empirical *P*<0.001; Fig. 5a), suggesting that they are subject to stochastic ASE. Next, we considered the 6,705 genes with an observed mean allelic ratio greater than 0.95. Of these, only 427 (6.4%) showed stochastic ASE not explained by technical noise (solid green line in Fig. 5a and Supplementary Data 1). We also observed that the fractions of SNPs showing ASE from the ‘T’ model (dotted red line in Fig. 5a) show good agreement with the observed values. These results suggest that much of the stochastic monoallelic expression observed in previous studies may not be of biological origin but rather technical variation.

When we simulated single-cell data allowing both technical and biological variability, we found that the simulated allelic ratios show good agreement with the observed values, except at high expression levels (Fig. 5b). Notice that the allelic ratios show a slight increase at an estimated number of transcripts per cell of ∼100 due to a few outlier genes that show monoallelic expression (Supplementary Fig. 11b,c). These results suggest that the genuine stochastic ASE can be explained by the biological cell-to-cell variability

Finally, we investigated the shared features of the 427 genes showing stochastic monoallelic expression not explained by technical noise. We found no enrichment of specific functional categories in these genes. In addition, they were not associated with specific transcription factor binding or histone modification patterns (Supplementary Note 8).

## Discussion

We provide a statistically sound framework to estimate biological noise by accounting for technical noise from scRNA-seq with the help of external RNA spike-ins. This framework enables us to investigate whether apparently abundant monoallelic expression observed in a single cell is driven by technical and biological noise. Intriguingly, we also show that the two alleles of a gene show correlated allelic expression across cells more often than expected by chance, potentially suggesting regulation by extrinsic factors (Supplementary Note 9).

Our noise decomposition approach is built upon two main ideas. First, cell-to-cell variability in capture and sequencing efficiency is allowed to account for the substantial differences in technical noise between cells. Second, by the general variance decomposition formula, the generative model enables us to decompose the observed total variance into technical and biological variance terms without strong parametric assumptions about the relationships between variation and gene expression and on the distributional form of the unobserved true number of mRNA molecules. In contrast to the deconvolution approach^10^, we do not assume that each transcript’s expression level is the sum of two independent, non-negative, counts that capture technical and biological processes. Consequently, our method does not underestimate the true expression level of a gene, thus yielding an improvement in estimating biological noise. It is also worth pointing out that the decomposition method is computationally more efficient than the deconvolution approach. Our approach allowed us, for the first time, to demonstrate that spike-ins could be used to statistically quantify the extent of stochastic ASE in scRNA-seq data. In contrast to some previous studies, we suggest that a substantial degree of stochastic ASE can be explained by technical noise.

More generally, it is essential to properly account for technical noise in scRNA-seq data as the high level of technical noise can easily skew biological interpretations of single-cell data. The most widely used approach to address this issue is to remove lowly expressed genes by employing a threshold on expression levels. In addition to the criticism that the choice of a threshold is arbitrary, determining what level of expression is enough to prevent misguided biological conclusions is highly dependent on biological questions, cell types and scRNA-seq protocols. The generative model that we present can instead be easily adapted to employ the simulation-based null hypothesis significance testing framework. The null distribution of a test statistic can be generated by simulating pseudo single cells assuming only technical variability, which is estimated from external RNA spike-in molecules. We can easily modify the generative model to simulate single-cell data in more complicated situations. To make this method more accessible, we provide an R function and the complete workflow to simulate single-cell data under various assumptions, which are available in the Supplementary Software. Our generative model, in principle, can be extended to a wide range of applications including identifying highly variable genes^12^, dissecting gene regulatory networks, inferring the kinetics of stochastic gene expression^21^ and detecting differentially expressed genes^22^.

## Methods

### Cell culture

G4 (C57BL/6Ncr x 129S6/SvEvTac) mouse hybrid^23^ cells were maintained on STO feeder cells. For the experiment, they were subcloned and cultured on gelatinized plates (Corning) in N2B27 basal media (NDiff 227, StemCells) with addition of 100 U ml^−1^ of recombinant human leukemia inhibitory factor (Millipore), 1 μM of Mek1/2 inhibitor (PD0325901, Stemgent) and 3 μM of GSK3β inhibitor (CHIR99021, Stemgent). After three passages, when cells reached 75% confluence they were harvested by trypsinizing them with 0.05% trypsin/EDTA (Gibco).

### RNA-seq

Single-cell libraries were prepared following the instructions in the Fluidigm manual ‘Using the C1 Single-Cell Auto Prep System to generate mRNA from Single Cells and Libraries for Sequencing’. Single cells were captured by loading cell suspension (1mln cells per ml) onto 10–17 microns Fluidigm C1 Single-Cell Auto Prep IFC. One microlitre of 1:100 dilution of ERCC RNA Spike-In Mix (Ambion) was added into lysis mix A to control for technical variation. Obtained cDNA was diluted to 0.1–0.3 ng μl^−1^ and sequencing libraries were prepared using Nextera XT DNA Sample Preparation Kit and the Nextera Index Kit (Illumina) according to the Fluidigm manual. Libraries from one chip were pooled, and paired-end 100 bp sequencing was performed on four lanes of an Illumina HiSeq2000 in the Wellcome Trust Sanger Institute.

### Mapping reads for gene-level counts

Paired-end reads were mapped to the *Mus musculus* genome (GRCm38) using GSNAP (version 2012-07-20) with default options^24^. From the GTF file of GRCm38 provided by Ensembl (release 73), we extracted known splice sites to detect splice junctions in reads and counted uniquely mapped reads for each gene using htseq-count^25^.

### Mapping reads for allele-level counts

We first took an intersection of SNPs present in the three 129 strains (129S1/SvImJ, 129S5/SvEvBrd and 129P2/OlaHsd) available in the Sanger mouse genomes project^26^ and then confirmed that the extracted SNPs do not overlap with a subline of C57BL/6 (C57BL/6NJ). From the resulting 5,038,206 SNPs between C57BL/6 and 129, we constructed the genome of the 129 strain by mutating nucleotides at the SNP positions. Paired-end reads were independently mapped to both the paternal (C57BL/6Ncr) and maternal (129S6/SvEvTac) genome using GSNAP with default options^24^. We discarded reads that are mapped to multiple locations. We extracted 120,036 exonic SNPs based on the GTF file of GRCm38 provided by Ensembl (release 73) and counted reads covering each exonic SNP from mpileup of samtools (version 0.1.19)^27^. We calculated two DESeq size factors for each cell, where one is from ERCC spike-ins (technical size factor capturing cell-to-cell variability in sequencing depth) and another is from endogenous genes (biological size factor capturing cell-to-cell variability in both sequencing depth and the total amount of mRNA molecules)^12^. Then, we normalized the raw read counts by dividing the counts by the corresponding size factors, that is, technical size factor for ERCC spike-ins and biological size factor for endogenous genes.

### Quality control on cells

We excluded cells from the downstream analysis if they satisfy the following criteria: (i) empty capture sites or capture sites with multiple cells or debris on the C1 chip by visual inspection under the microscope, (ii) cells that have fewer than 500,000 (or 50%) reads mapped to exons (indicator for sequencing library failure), (iii) cells that have greater than 10% reads mapped to 37 genes on the mitochondrial chromosome (indicator for cell rupture during the process of microfluidic cell capture^13^).

### Quality control on SNPs

To exclude SNPs affected by the systematic bias towards the reference allele, we applied the following criteria: (i) SNPs not annotated as ‘PASS’ in the FILTER column of the VCF file provided by the Mouse Genomes Project (version 3) were removed, (ii) SNPs that have a number of other SNP sites located within the SNP-flanking region(± read length) larger than or equal to 4 were removed, (iii) SNPs with at least one indel within the SNP-flanking region were removed^28^, (iv) SNPs on chromosome X (the mouse used is male) were removed.

### Noise decomposition method

The underlying generative model of our noise decomposition method is described in Supplementary Note 1 and numerical procedures for estimating parameters are provided in Supplementary Note 2. A statistical framework for decomposing the total variance into the technical and biological variance based on the generative model is described in Supplementary Note 3. The R code and the complete workflow to run the R code are available in Supplementary Software.

## Additional information

**How to cite this article:** Kim, J. K. *et al.* Characterizing noise structure in single-cell RNA-seq distinguishes genuine from technical stochastic allelic expression. *Nat. Commun.* 6:8687 doi: 10.1038/ncomms9687 (2015).

**Accession code:** Sequencing data are available in the ArrayExpress database under accession number E-MTAB-2600.

## Supplementary Material

Supplementary Information Supplementary Figures 1-14, Supplementary Notes 1-9 and Supplementary References.

Supplementary Data 1 Genes showing stochastic allele specific expression not explained by technical noise

Supplementary Software Ancillary files and scripts that are necessary to run the code.
