# Corrigendum: Characterizing noise structure in single-cell RNA-seq distinguishes genuine from technical stochastic allelic expression

**DOI:** 10.1038/ncomms10415

**Published:** 2016-01-11

**Authors:** Jong Kyoung Kim, Aleksandra A. Kolodziejczyk, Tomislav Ilicic, Sarah A. Teichmann, John C. Marioni

Nature Communications 6: Article number: 868710.1038/ncomms9687 (2015); Published: 10222015; Updated: 01112016.

The original version of this Article contained an error in the spelling of the author Tomislav Ilicic, which was incorrectly given as Tomislav Illicic. This has now been corrected in both the PDF and HTML versions of the Article.

