# Supplementary material for: Characterizing noise structure in single-cell RNA-seq distinguishes genuine from technical stochastic allelic expression
Source: Nat Commun. 2015 Oct 22;6:8687. doi: 10.1038/ncomms9687 (PMC4627577; doi:10.1038/ncomms9687)
Supplement: Supplementary Information — Supplementary Figures 1-14, Supplementary Notes 1-9 and Supplementary References. [file ncomms9687-s1.pdf]

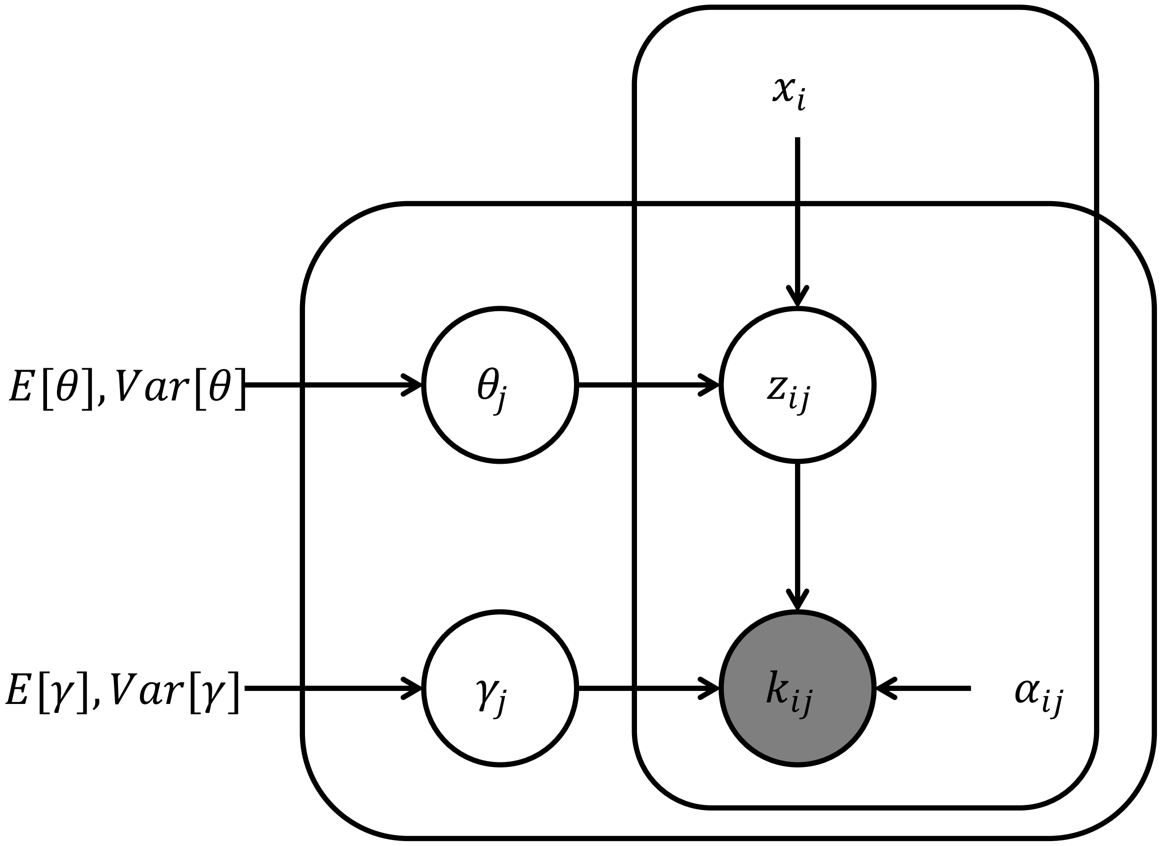

Supplementary Figure 1: Graphical representation of the generative model.

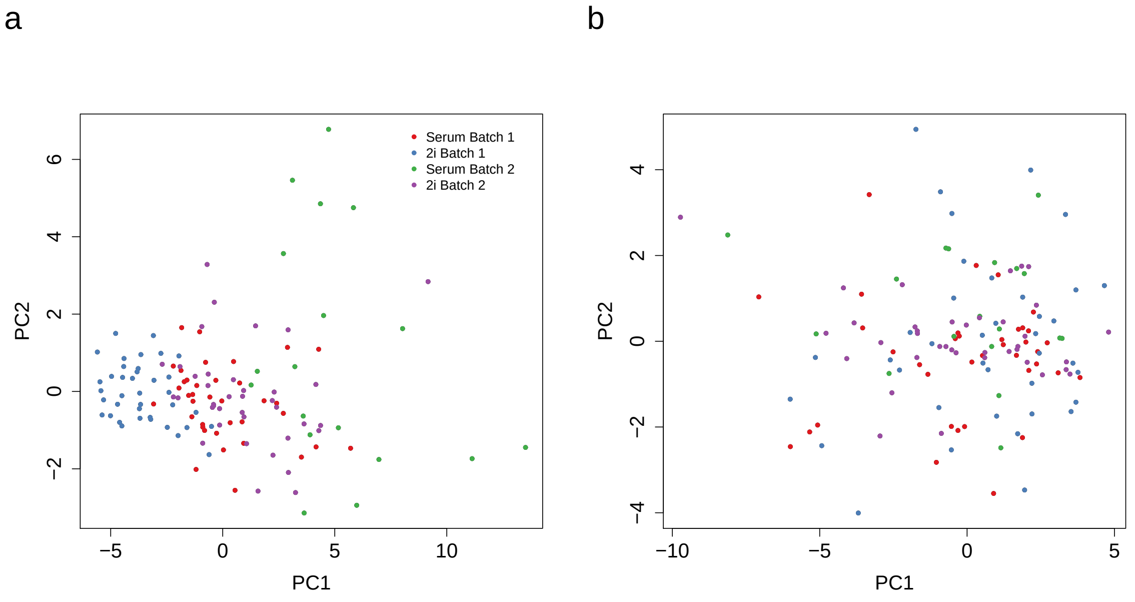

Supplementary Figure 2: Score plot of the first two principal components for 131 cells of Grün *et al.*[1] using ERCC spike-ins. Batches are indicated by colors. (a) Before normalization. (b) After normalization.

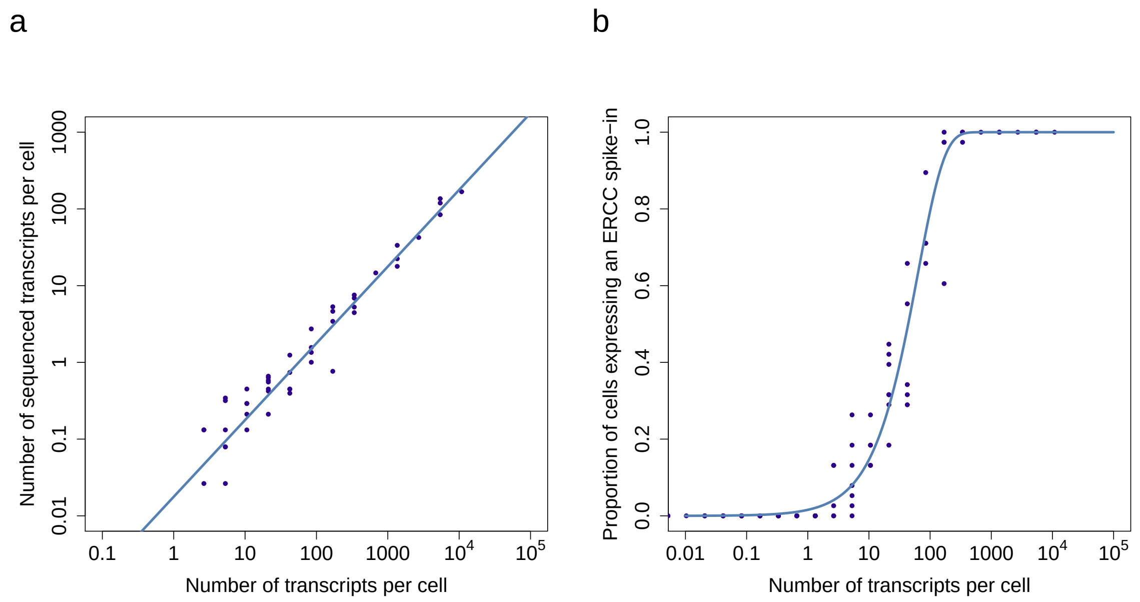

Supplementary Figure 3: Estimating $E[\gamma]$ and $E[\theta]$ from ERCC spike-ins in the first batch of 2i-grown mESCs of Grün *et al.*[1]. (a) The number of sequenced transcripts per cell is plotted against the number of added transcripts per cell. Dots correspond to ERCC spike-ins. The solid line represents the linear fit between the expected number of sequenced transcripts and the number of added transcripts per cell. (b) The proportion of cells having non-zero read counts of ERCC spike-ins versus the number of added transcripts per cell. The solid line represents the expected proportion of cells having non-zero read counts of ERCC spike-ins as a function of the number of added transcripts per cell.

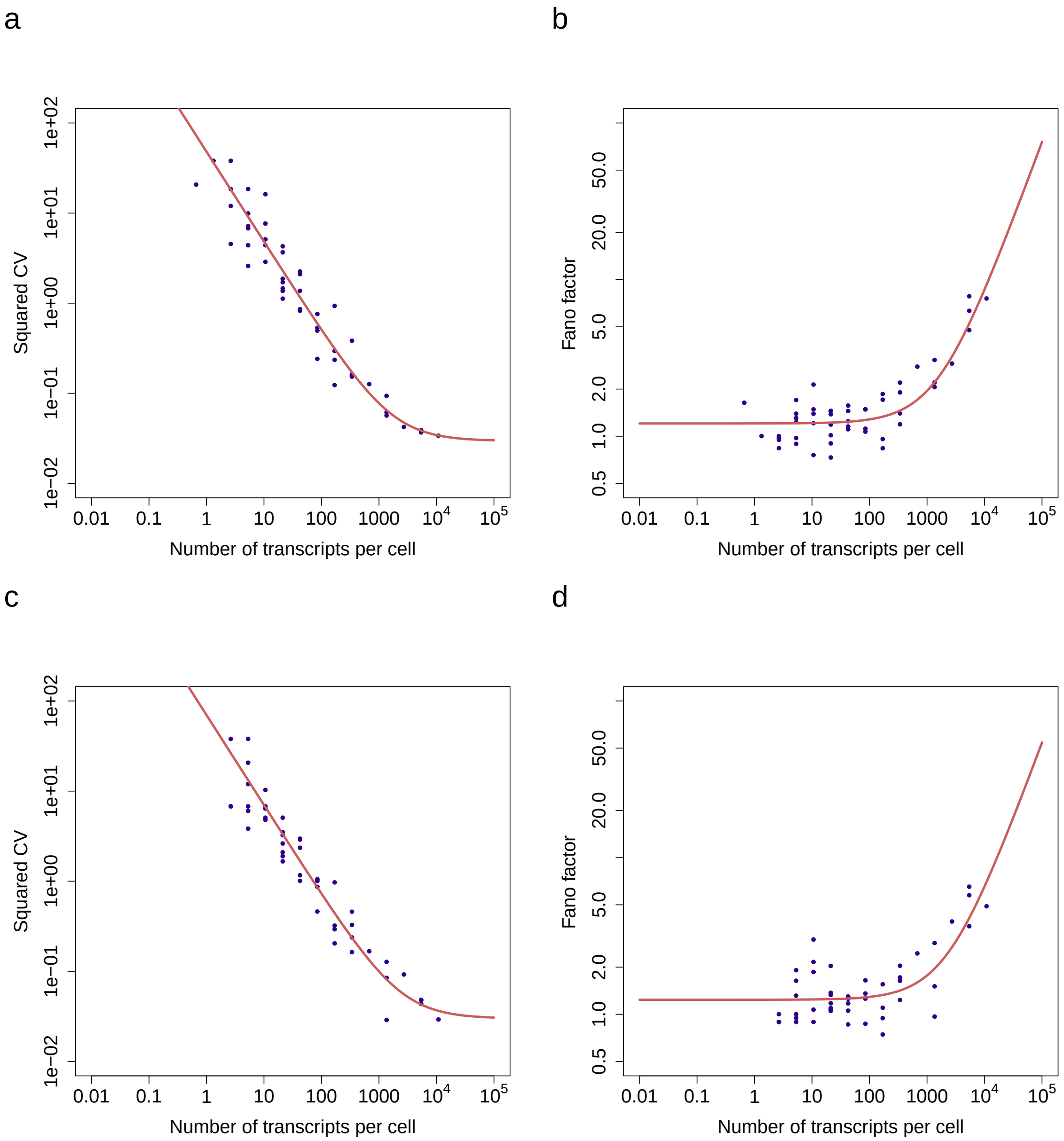

Supplementary Figure 4: Estimating Var[γ] and Var[θ] from ERCC spike-ins in the first batch of serum-grown (a,b) or 2i-grown (c,d) mESCs. Squared CV (a,c) and Fano factor (b,d) are plotted against the number of added transcripts of ERCC spike-ins per cell. Dots correspond to ERCC spike-ins and the solid line represents the technical noise fit.

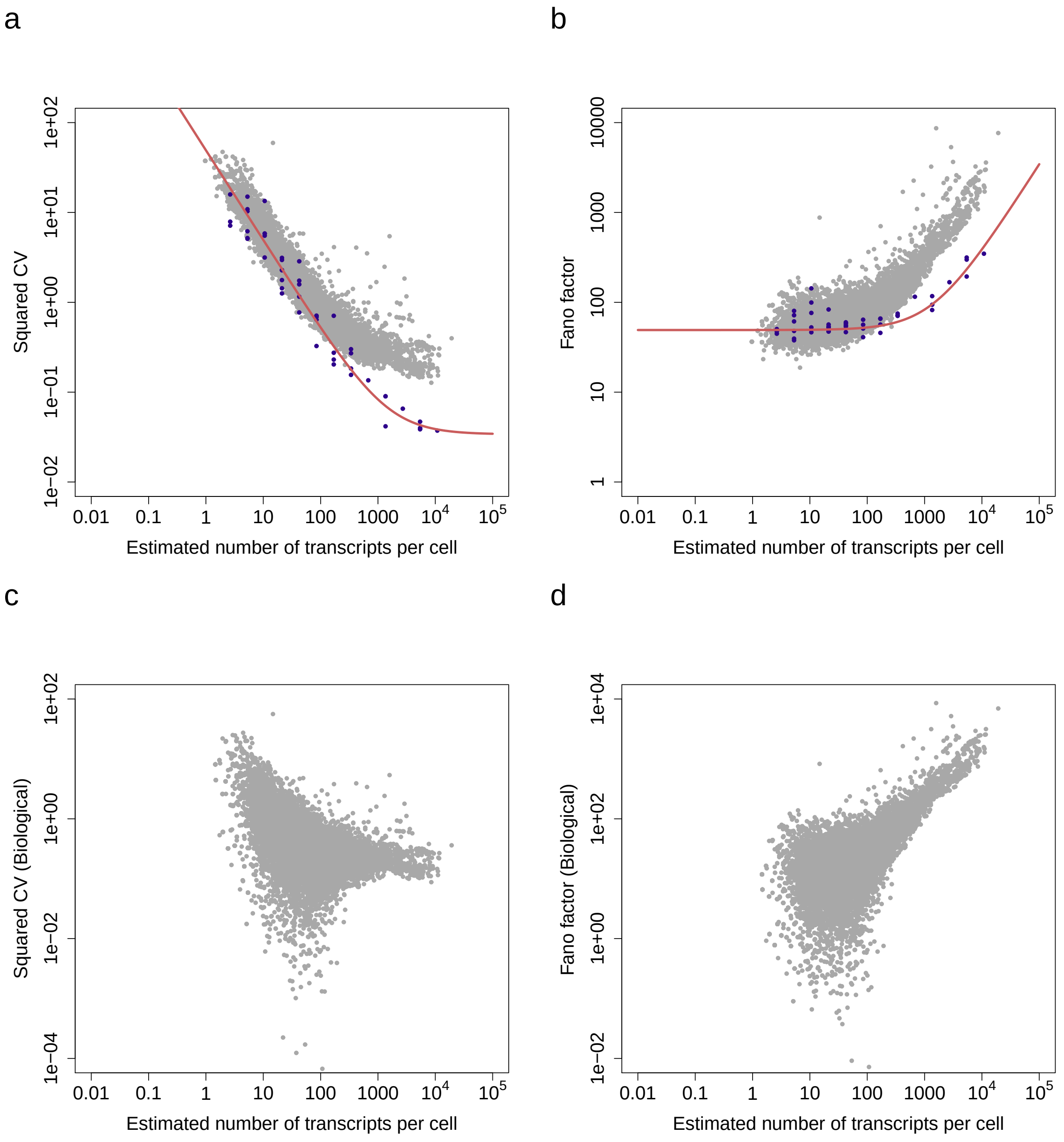

Supplementary Figure 5: Technical noise fit and inference of biological noise using ERCC spike-ins in 2i-grown mESCs. Squared CV (a) and Fano factor (b) are plotted against the estimated number of transcripts per cell in 2i-grown mESCs. Gray dots correspond to mouse genes and blue dots to ERCC spike-ins. The solid red line represents the technical noise fit. Estimated squared CV (c) and Fano factor (d) of biological noise are plotted against the estimated number of transcripts per cell.

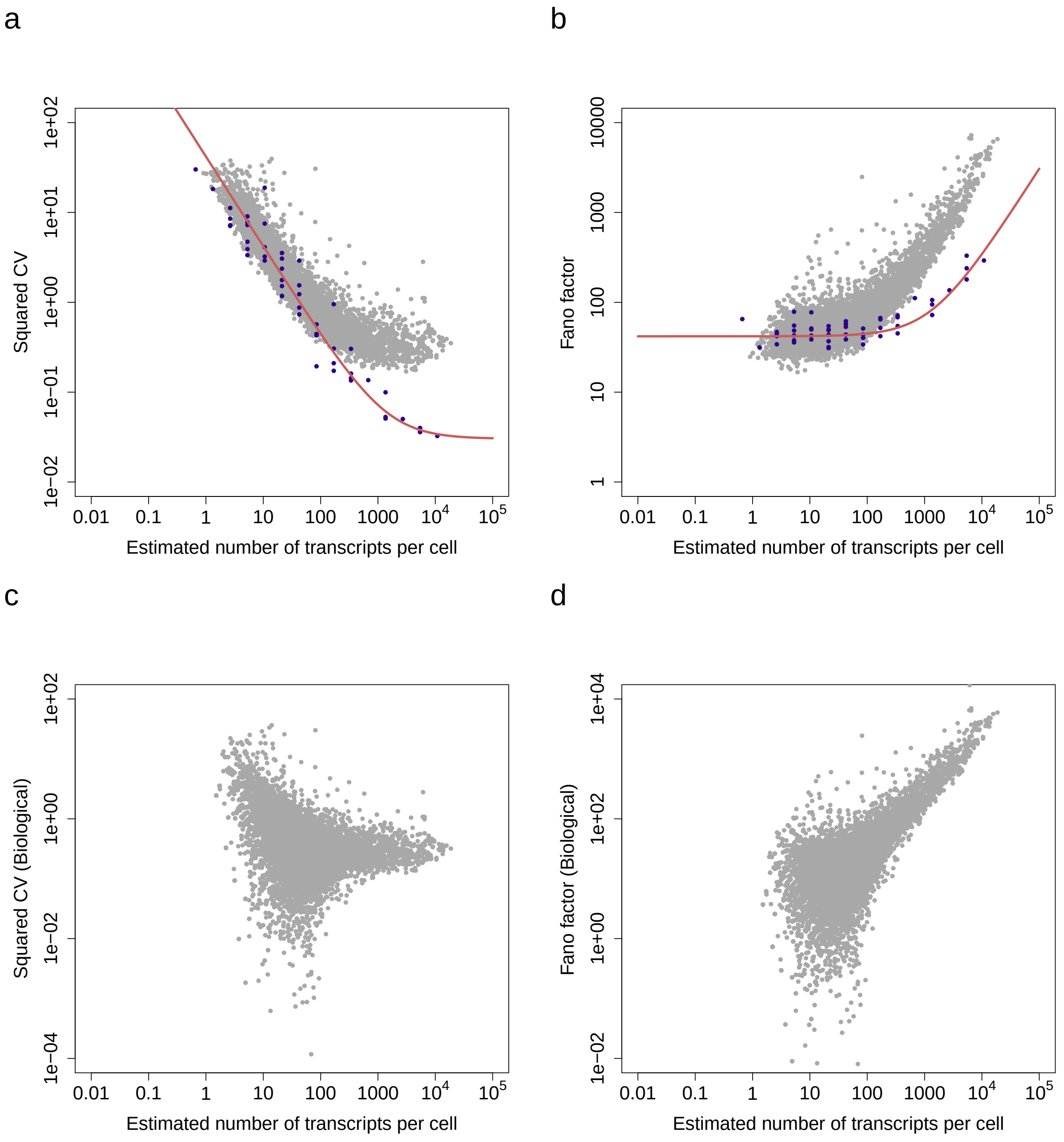

Supplementary Figure 6: Technical noise fit and inference of biological noise using ERCC spike-ins in serum-grown mESCs. Squared CV (a) and Fano factor (b) are plotted against the estimated number of transcripts per cell. Gray dots correspond to mouse genes and blue dots to ERCC spike-ins. The solid red line represents the technical noise fit. Estimated squared CV (c) and Fano factor (d) of biological noise are plotted against the estimated number of transcripts per cell.

a

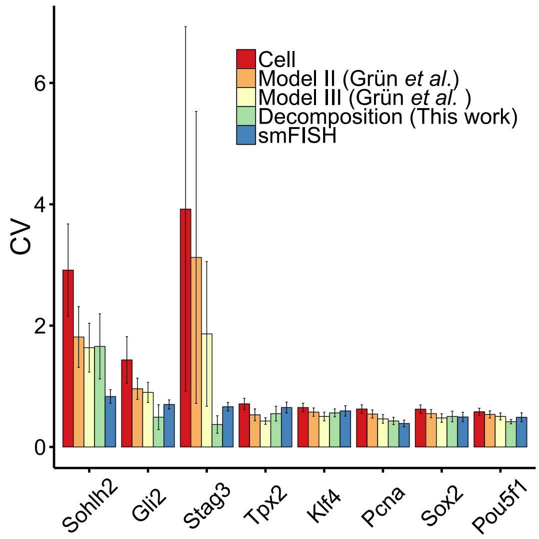

Supplementary Figure 7: Validation of estimated biological noise of serum-grown mESCs by single molecule FISH. Bar plot depicts the measured CV (y-axis) of chosen genes by each method: total noise by scRNA-seq (Cell); Model II and III of Grün *et al.*[1]; our noise decomposition method (Decomposition); single cell FISH (smFISH). Genes chosen by Grün *et al.*[1] to cover a dynamic range of gene expression are sorted by their expression levels: lowly expressed genes (*Sohlh2*, *Notch1*, *Gli2* and *Stag3*), moderately expressed genes (Tpx2), and highly expressed genes (*Pou5f1*, *Sox2*, *Pcna2* and *Klf4*). *Notch1* is not available in serum-grown mESCs of Grün *et al.*[1]. Error bars represent standard deviation (sd): bootstrap sd for our predictions; sd derived from estimated standard errors of the parameters of a negative binomial distribution for other methods.

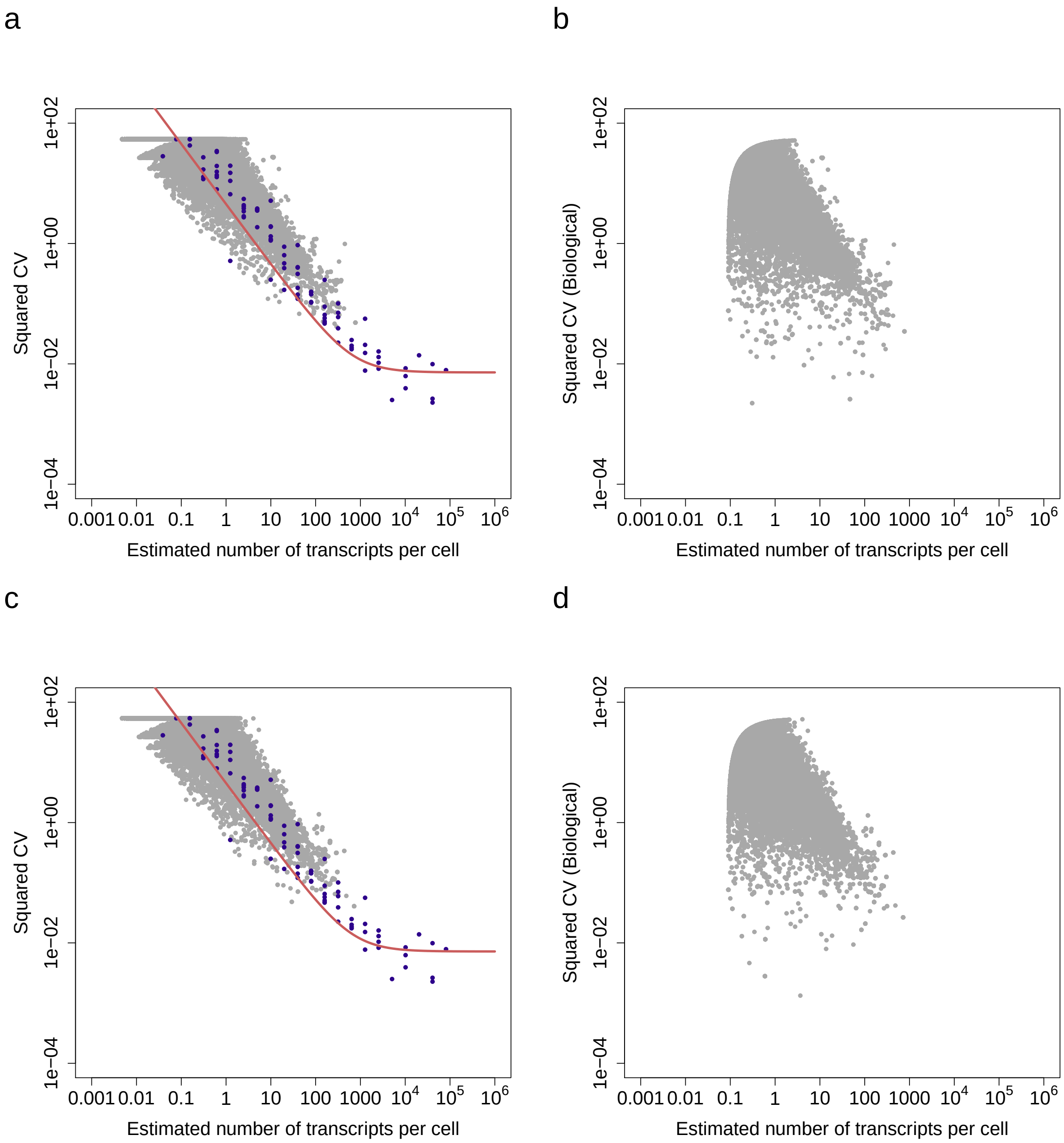

Supplementary Figure 8: Technical noise fit and inference of biological noise of both paternal and maternal alleles in 2i-grown mESCs. Squared CV of paternal alleles (a) and maternal alleles (c) versus the estimated number of transcripts per cell. Gray dots correspond to mouse genes and blue dots to ERCC spike-ins. The solid red line represents the technical noise fit. Estimated biological squared CV of paternal alleles (b) and maternal alleles (d) are plotted against the estimated number of transcripts per cell.

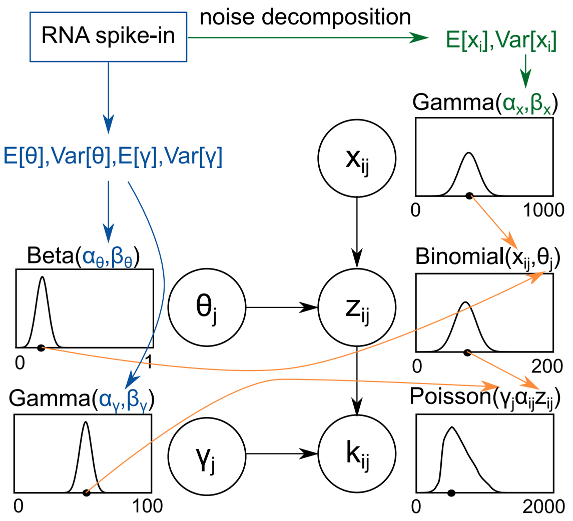

Supplementary Figure 9: Schematic representation of simulating single-cell data. Our goal is to draw read counts k_{ij} assuming only technical noise or both technical and biological noise. To do this, we first estimate the expectation and variance of θ_j (capture efficiency of cell j) and γ_j (sequencing efficiency of cell j) from the external RNA spike-in molecules. The four parameters (colored in blue) are used to estimate parameters of beta (θ_j) and gamma (γ_j) distributions. By decomposing the total observed variance, we can estimate the expectation and variance of x_{ij} (unobserved number of RNA molecules of gene i in cell j, colored in green) that are again used to estimate parameters of a gamma distribution for x_{ij}. If we assume only technical noise, we set the variance of x_{ij} to 0. We start with x_{ij} and draw a sample from the gamma distribution. We then draw samples (plotted as black circles) in order (x_{ij}, θ_j, z_{ij}, γ_j, and k_{ij}). The samples drawn from parent variables (e.g. x_{ij} and θ_j are parent variables of z_{ij}) are used as parameters of their child variables (plotted as orange arrows).

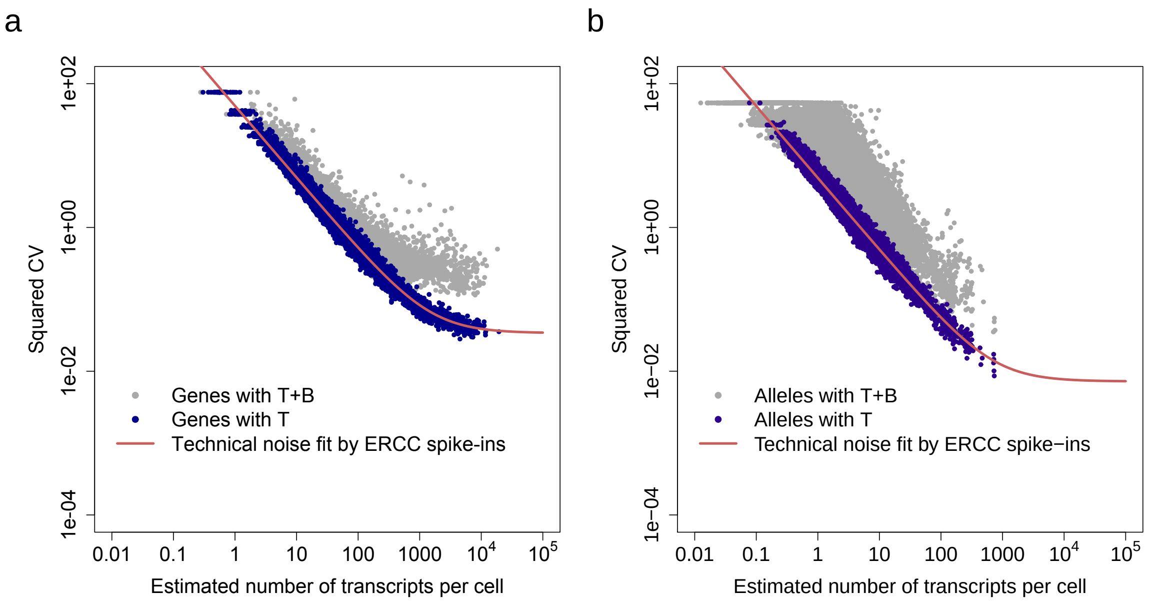

Supplementary Figure 10: Simulated single-cell data reflecting the real scRNA-seq data. Squared CV of simulated genes in 2i-grown mESCs using the UMI protocol (a) and of simulated alleles in 2i-grown mESCs using the full length protocol (b) are plotted against the estimated number of transcripts per cell. Gray dots correspond to genes or alleles simulated assuming both technical and biological noise ("T+B") and blue dots to genes or alleles simulated assuming only technical noise ("T"). The solid red line represents the technical noise fit by ERCC spike-ins.

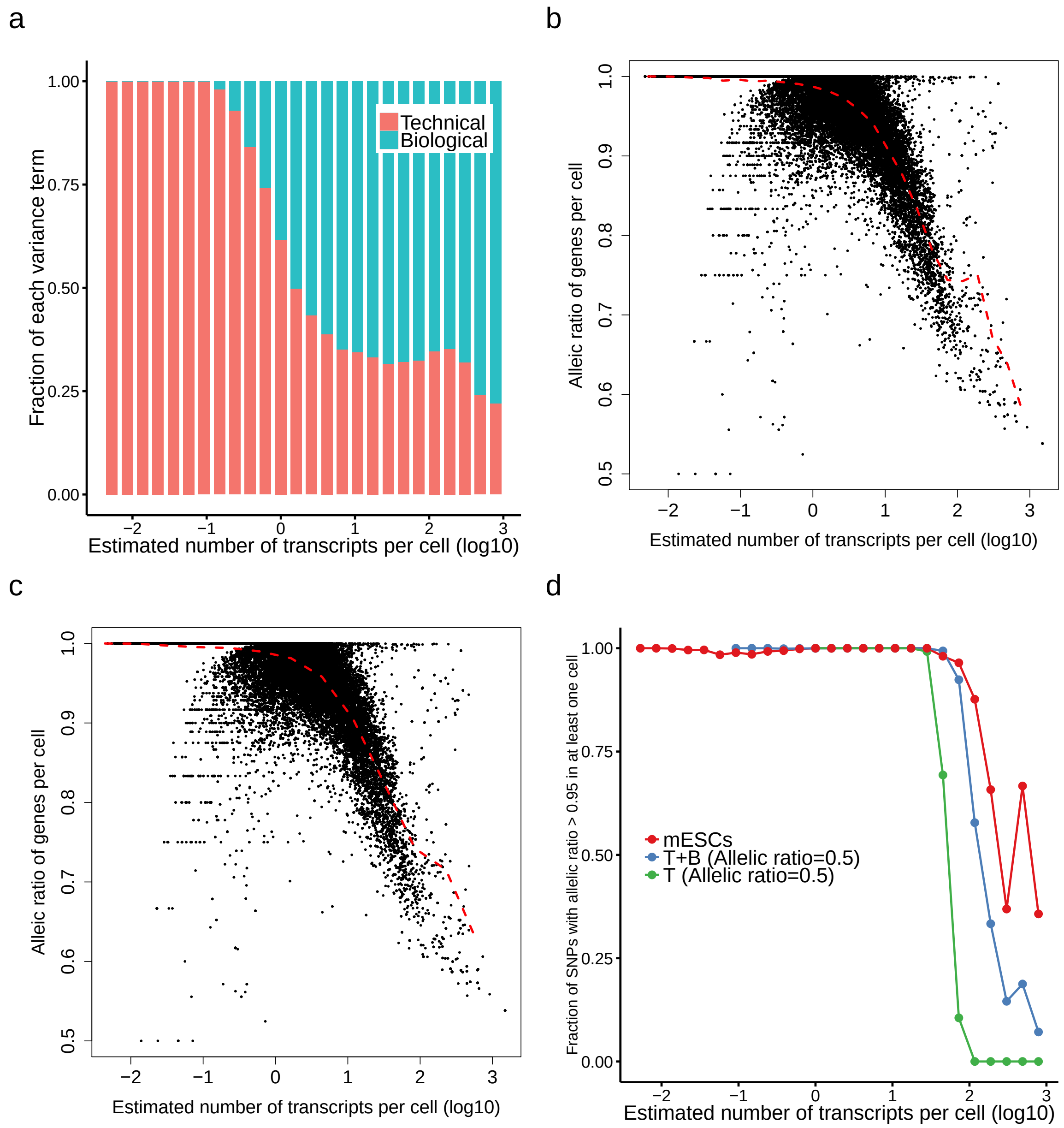

Supplementary Figure 11: (a) Mean fraction of technical and biological variance binned by expression levels. (b) Fraction of most expressed alleles for SNPs per cell is plotted against the estimated number of transcripts per cell. The dotted red line represents the mean fraction of most expressed alleles for SNPs binned by expression levels. The number of bins is 30. (c) When we decreased the binning resolution, the spike between 1.5 and 2.5 disappeared. The number of bins is 15. (d) Mean fraction of expressed SNPs with allelic ratio larger than 0.95 in at least one cell. Colors indicate different approaches for computing the mean fraction by scRNA-seq measurements (mESCs), "T" model with an allelic ratio fixed to 0.5, and "T+B" model with an allelic ratio fixed to 0.5.

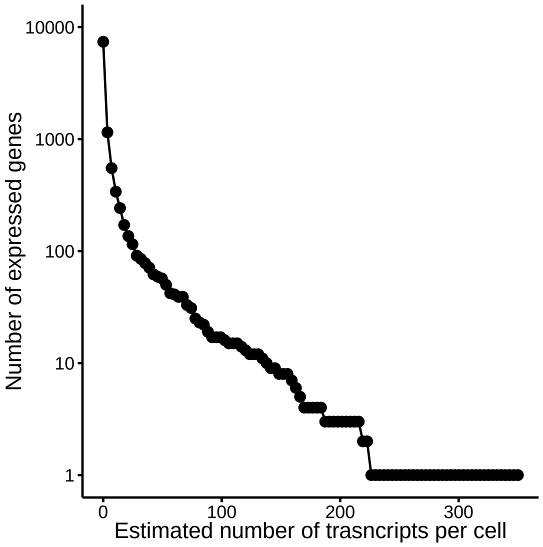

Supplementary Figure 12: Number of expressed genes versus the expression cutoff. The number of expressed genes is defined by the number of genes with the estimated number of transcripts per cell larger than the given cutoff for at least one allele.

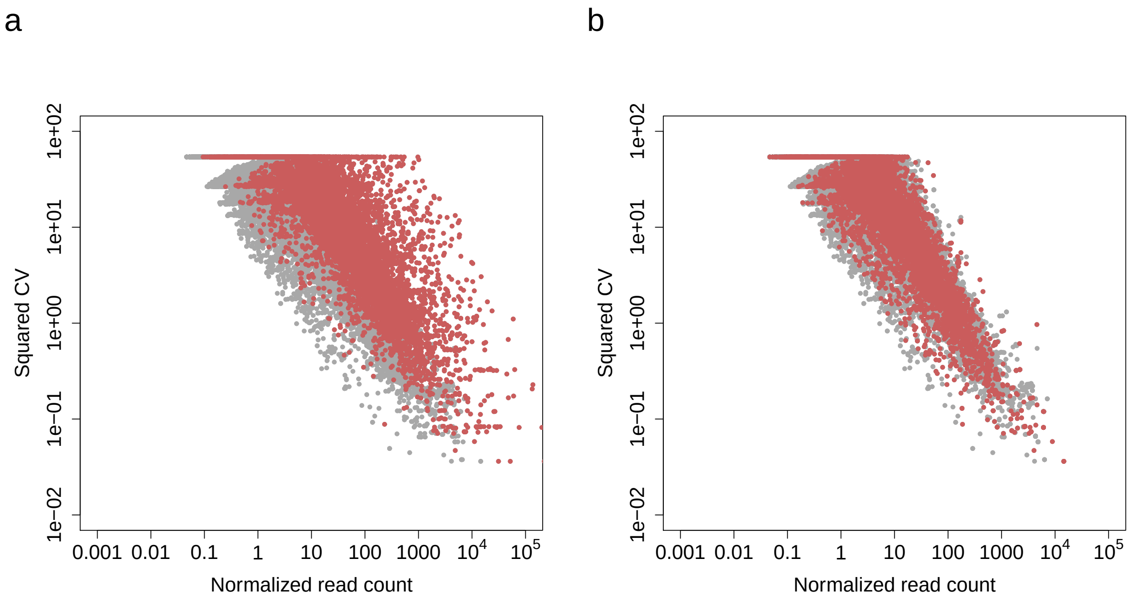

Supplementary Figure 13: Overestimation of the expression levels of SNPs near the starts and ends of annotated genes. (a) Squared CV of the sum of normalized read count by α_{ij} mapped to both paternal and maternal alleles versus the mean normalized read count. Red dots correspond to SNPs with their effective lengths smaller than the length of reads (100 in this study). (b) The effective lengths of SNPs near the starts and ends of annotated genes are set to the length of reads (100).

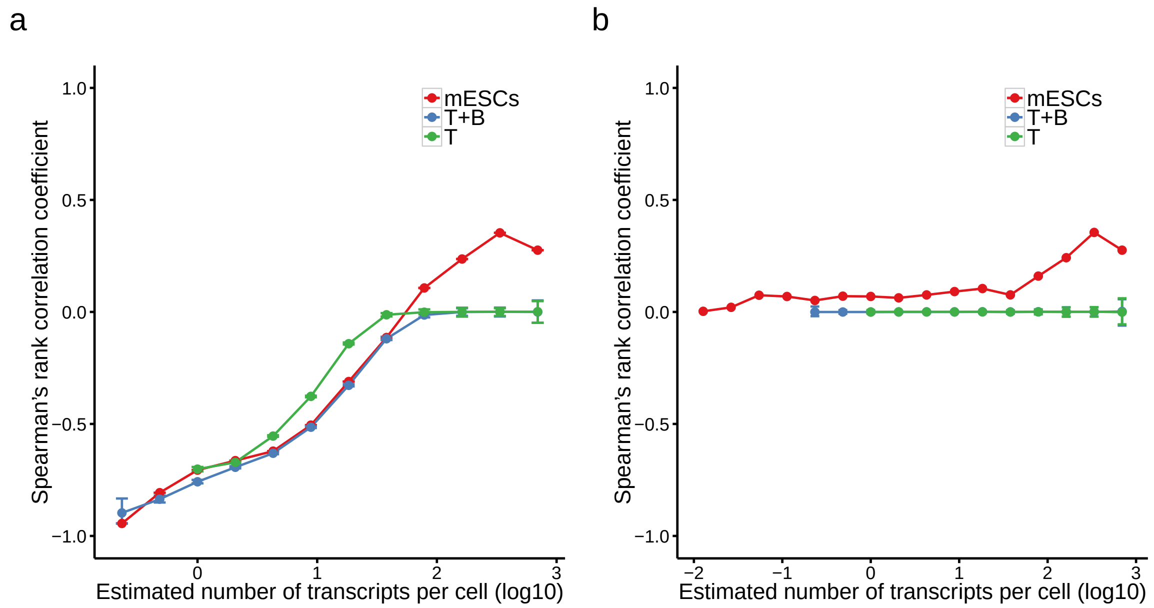

Supplementary Figure 14: Mean Spearman's rank correlation coefficients for SNPs binned by expression levels with colors as in Fig. 5b. (a) For each SNP, cells with the estimated number of transcripts covering both alleles fewer than 5 were not included. (b) We did not filter out cells with the estimated number of transcripts covering both alleles fewer than 5. Error bars denote 95% CIs by bootstrap (100 bootstrap samples).

Supplementary Note 1: A generative model for technical noise

Let k_{ij} be the observed read count of spike-in RNA molecule i in cell j. k_{ij} can be the number of sequenced transcripts if unique molecular identifiers (UMIs) are used. Assuming that each cell's lysate is spiked with a mixture of synthetic RNAs of known concentration, the observed count k_{ij} can be generated by the following model:

1. The number of transcripts available for sequencing following reverse transcription and cDNA amplification for spike i in cell j,z_{ij}, is selected from a binomial distribution:

   $$z_{ij} \sim \text{Binomial}(z_{ij}|x_i, \theta_j)$$

   where x_i is the known number of added spike-in transcripts and θ_j is the capture efficiency for cell j. The capture efficiency explains the stochastic RNA loss during the sample preparation procedure prior to sequencing. It should be noted that we do not model cell lysis inefficiencies since the external RNA spike-ins are added after cell lysis.

2. Given z_{ij}, the observed read count is Poisson distributed such that

   $$k_{ij} \sim \text{Poisson}(k_{ij}|\gamma_j \alpha_{ij} z_{ij})$$

   where γ_j is the amplification factor (or sequencing efficiency) of cell j converting the number of transcripts to the observed read count, and α_{ij} is the product of the length of spike-in RNA i measured in kilobase and the normalization factor for cell j estimated

using DESeq[2]. If k_{ij} is the number of sequenced transcripts obtained by UMIs, α_{ij} is equal to 1. γ_j is still needed to reflect cell-to-cell variability in sequencing efficiency.

To allow cell-to-cell variability in the capture efficiency and amplification factor, γ_j and θ_j are treated as random variables and assumed to be independent, but we do not assume a distribution of a specific form. More formally, the γ_j are independently and identically distributed with finite mean and variance such that

$$E[\gamma_j] = E[\gamma]$$
$$\text{Var}[\gamma_j] = \text{Var}[\gamma]$$

The same assumptions apply to θ_j.

Supplementary Note 2: Estimating parameters

We estimate the four parameters $E[\gamma], \text{Var}[\gamma], E[\theta]$ and $\text{Var}[\theta]$ using the observed ERCC spike-in counts. We first estimate the expected value of the product of γ and θ, $E[\eta] = E[\gamma]E[\theta]$, by relating the sample mean of k_{ij} to a linear function of x_i. Letting $k_i = \frac{1}{M}\sum_{j=1}^{M}\frac{k_{ij}}{\alpha_{ij}}$, the expected value of k_i is given by

$$E[k_i] = E[\gamma]E[\theta]x_i$$

Suppose that there exist N spike-in molecules, $\{(x_i, k_i)\}_{i=1}^{N}$, which are independent and identically distributed. The linear least squares estimate $\widehat{E[\eta]}$ is the solution to

$$\min_{E[\eta]} \sum_{i=1}^{N} (k_i - E[\eta]x_i)^2 \tag{1}$$

where we have a closed form solution

$$\widehat{E[\eta]} = \frac{\sum_{i=1}^{N} k_i x_i}{\sum_{i=1}^{N} x_i^2}.$$

Second, $E[\gamma]$ and $E[\theta]$ can be separately estimated by noting the nonlinear relation between the proportion of cells having non-zero read counts and x_i. Letting $p_i = \frac{1}{M}\sum_{j=1}^{M} 1(k_{ij} > 0)$, we obtain

$$\begin{aligned} E[p_i] &= 1 - \frac{1}{M}\sum_{j=1}^{M} E_{z_{ij},\theta_j,\gamma_j}[e^{-\gamma_j \alpha_{ij} z_{ij}}] \\ &= 1 - \frac{1}{M}\sum_{j=1}^{M} E_{\theta_j,\gamma_j}\left[\left(1-\theta_j+\theta_j e^{-\gamma_j\alpha_{ij}}\right)^{x_i}\right] \\ &\approx 1 - \frac{1}{M}\sum_{j=1}^{M}\left(1-E[\theta]+E[\theta]e^{-\frac{E[\eta]}{E[\theta]}\alpha_{ij}}\right)^{x_i}. \end{aligned}$$

where we use first-order Taylor approximation in (γ_j, θ_j). Plugging the least squares estimate $\widehat{E[\eta]}$ into the above equation, the nonlinear least squares estimate $\widehat{E[\theta]}$ (and $\widehat{E[\gamma]}$ by dividing $\widehat{E[\eta]}$ by $\widehat{E[\theta]}$) can be obtained by solving the following constrained optimization problem

$$\begin{aligned} \min_{E[\theta]} \quad & \sum_{i=1}^{N}\left(p_i - \left(1 - \frac{1}{M}\sum_{j=1}^{M}\left(1-E[\theta]+E[\theta]e^{-\frac{\widehat{E[\eta]}}{E[\theta]}\alpha_{ij}}\right)^{x_i}\right)\right)^2 \\ \text{subject to} \quad & 0 \le E[\theta] \le 1. \end{aligned} \tag{2}$$

We use the *nlsLM* function (a modification of the Levenberg-Marquardt algorithm) in the *minpack.lm* R package by setting the lower bound and upper bound of $E[\theta]$ to 0 and 1, respectively.

Finally, we estimate the remaining parameters $\text{Var}[\gamma]$ and $\text{Var}[\theta]$ based on the Fano factor. By the general variance decomposition formula[3], the variance of k_{ij} can be decomposed

into

$$\mathrm{Var}[k_{ij}] = \overbrace{E[\mathrm{Var}[k_{ij}|z_{ij},\theta_j,\gamma_j]]}^{\text{shot noise}} + \overbrace{E[\mathrm{Var}[E[k_{ij}|z_{ij},\theta_j,\gamma_j]|\theta_j,\gamma_j]]}^{\text{variation generated by } z_{ij}}$$
$$+ \overbrace{E[\mathrm{Var}[E[k_{ij}|\theta_j,\gamma_j]|\gamma_j]]}^{\text{variation generated by } \theta_j} + \overbrace{\mathrm{Var}[E[k_{ij}|\gamma_j]]}^{\text{variation generated by } \gamma_j}$$

The first term explains the variation coming from sources other than z_{ij}, θ_j and γ_j, which corresponds to Poisson noise (or shot noise) in this model and is given by

$$E[\mathrm{Var}[k_{ij}|z_{ij},\theta_j,\gamma_j]] = E[\gamma]E[\theta]\alpha_{ij}x_i$$

The second term quantifies the variation generated by the stochastic RNA loss during the sample preparation procedure (not cell lysis), which is given by

$$E[\mathrm{Var}[E[k_{ij}|z_{ij},\theta_j,\gamma_j]|\theta_j,\gamma_j]] = \left(\mathrm{Var}[\gamma] + E[\gamma]^2\right)\alpha_{ij}^2 x_i \left(E[\theta] - \left(\mathrm{Var}[\theta] + E[\theta]^2\right)\right)$$

The third term quantifies the variation generated by fluctuations in capture efficiency θ_j between cells, which follows from

$$E[\mathrm{Var}[E[k_{ij}|\theta_j,\gamma_j]|\gamma_j]] = E[\mathrm{Var}[k_{ij}|\gamma_j] - \mathrm{Var}[k_{ij}|\theta_j,\gamma_j]]$$

by the law of total conditional variance[3]

$$\mathrm{Var}[k_{ij}|\gamma_j] = E[\mathrm{Var}[k_{ij}|\theta_j,\gamma_j]|\gamma_j] + \mathrm{Var}[E[k_{ij}|\theta_j,\gamma_j]|\gamma_j]$$

This term is given by

$$E[\mathrm{Var}[E[k_{ij}|\theta_j,\gamma_j]|\gamma_j]] = \left(\mathrm{Var}[\gamma] + E[\gamma]^2\right)\mathrm{Var}[\theta]\alpha_{ij}^2 x_i^2$$

The last term quantifies the variation attributable to cell-to-cell fluctuations in the amplification factor γ_j, which is given by

$$\mathrm{Var}[E[k_{ij}|\gamma_j]] = \mathrm{Var}[\gamma]E[\theta]^2\alpha_{ij}^2 x_i^2$$

For convenience, we compute the variance of $\frac{k_{ij}}{\alpha_{ij}}$

$$\begin{aligned}\mathrm{Var}\left[\frac{k_{ij}}{\alpha_{ij}}\right] &= \left(\frac{E[\gamma]E[\theta]}{\alpha_{ij}} + \left(\mathrm{Var}[\gamma] + E[\gamma]^2\right)\left(E[\theta] - \left(\mathrm{Var}[\theta] + E[\theta]^2\right)\right)\right) x_i \\ &\quad + \left(\left(\mathrm{Var}[\gamma] + E[\gamma]^2\right)\mathrm{Var}[\theta] + \mathrm{Var}[\gamma]E[\theta]^2\right) x_i^2\end{aligned}$$

$$\begin{aligned}CV^2\left[\frac{k_{ij}}{\alpha_{ij}}\right] &= \left(\frac{1}{E[\gamma]E[\theta]\alpha_{ij}} + \frac{\left(\mathrm{Var}[\gamma] + E[\gamma]^2\right)}{E[\gamma]^2E[\theta]^2}\left(E[\theta] - \left(\mathrm{Var}[\theta] + E[\theta]^2\right)\right)\right)\frac{1}{x_i} \\ &\quad + \frac{\left(\mathrm{Var}[\gamma] + E[\gamma]^2\right)\mathrm{Var}[\theta] + \mathrm{Var}[\gamma]E[\theta]^2}{E[\gamma]^2E[\theta]^2}\end{aligned}$$

$$\begin{aligned}\mathrm{F}\left[\frac{k_{ij}}{\alpha_{ij}}\right] &= \frac{1}{\alpha_{ij}} + \frac{\left(\mathrm{Var}[\gamma] + E[\gamma]^2\right)}{E[\gamma]E[\theta]}\left(E[\theta] - \left(\mathrm{Var}[\theta] + E[\theta]^2\right)\right) \\ &\quad + \frac{\left(\mathrm{Var}[\gamma] + E[\gamma]^2\right)\mathrm{Var}[\theta] + \mathrm{Var}[\gamma]E[\theta]^2}{E[\gamma]E[\theta]} x_i\end{aligned}$$

Let w_i be the sample variance for spike-in i based upon the normalized read count $\frac{k_{ij}}{\alpha_{ij}}$ such that

$$w_i = \frac{1}{M-1}\sum_{j=1}^{M}\left(\frac{k_{ij}}{\alpha_{ij}} - k_i\right)^2$$

Based on the expectation of the sample variance w_i and sample mean k_i, the Fano factor can be derived independently of the cell index j

$$\begin{aligned}\mathrm{F}[i] &= \frac{E[w_i]}{E[k_i]} = \beta_i + \frac{\left(\mathrm{Var}[\gamma] + E[\gamma]^2\right)}{E[\gamma]}\left(1 - \frac{\left(\mathrm{Var}[\theta] + E[\theta]^2\right)}{E[\theta]}\right) \\ &\quad + \frac{\left(\mathrm{Var}[\gamma] + E[\gamma]^2\right)\mathrm{Var}[\theta] + \mathrm{Var}[\gamma]E[\theta]^2}{E[\gamma]E[\theta]} x_i\end{aligned}$$

where $\beta_i = \frac{1}{M}\sum_{j=1}^{M}\frac{1}{\alpha_{ij}}$. Plugging the nonlinear least squares estimates of $E[\gamma]$ and $E[\theta]$ into $F[i]$, we then estimate $\mathrm{Var}[\gamma]$ and $\mathrm{Var}[\theta]$ by solving the following nonlinear least squares problem

$$\begin{aligned}\min_{\mathrm{Var}[\gamma],\mathrm{Var}[\theta]} \quad & \sum_{i=1}^{N}\left(\frac{w_i}{k_i} - F[i]\right)^2 \\ \text{subject to} \quad & \mathrm{Var}[\gamma], \mathrm{Var}[\theta] \geq 0\end{aligned} \tag{3}$$

We use the *nlsLM* function in *minpack.lm* R package by setting the lower bound of Var$[\gamma]$ and Var$[\theta]$ to 0.

Supplementary Note 3: Decomposing the total variance into the technical and biological variance

We are interested in estimating the biological variance from the variance of observed read counts whilst accounting for the technical variance. Our proposed generative model provides a statistically sound framework for this purpose with no further assumptions on the form of distributions of observed read counts or the number of transcripts in a single cell.

Suppose that x_{ij} (the number of transcripts of gene i in cell j) is a random variable with mean μ_i and variance σ_i^2. It should be noted that x_{ij} is allowed to vary across cells, which allows biological noise to be incorporated. The expectation of k_{ij} is given by

$$E[k_{ij}] = E[\gamma]E[\theta]\alpha_{ij}\mu_i$$

where $\alpha_{ij} = 1$ for tag-based scRNA-seq experiments. For the length of SNPs, see Supplementary Note 7.

By the general variance decomposition formula, the variance of k_{ij} can be decomposed

into

$$\begin{aligned}
\mathrm{Var}[k_{ij}] \;&=\; \overbrace{E[\mathrm{Var}[k_{ij}|z_{ij},\theta_j,\gamma_j,x_{ij}]]}^{\text{shot noise}} + \overbrace{E[\mathrm{Var}[E[k_{ij}|z_{ij},\theta_j,\gamma_j,x_{ij}]|\theta_j,\gamma_j,x_{ij}]]}^{\text{variation generated by } z_{ij}} \\
&+ \overbrace{E[\mathrm{Var}[E[k_{ij}|\theta_j,\gamma_j,x_{ij}]|\gamma,\theta]]}^{\text{biological noise}} + \overbrace{E[\mathrm{Var}[E[k_{ij}|\gamma_j,\theta_j]|\gamma_j]]}^{\text{variation generated by } \theta_j} \\
&+ \overbrace{\mathrm{Var}[E[k_{ij}|\gamma_j]]}^{\text{variation generated by } \gamma_j}
\end{aligned}$$

where

$$\begin{aligned}
E[\mathrm{Var}[k_{ij}|z_{ij},\theta_j,\gamma_j,x_{ij}]] &= E[\gamma]E[\theta]\alpha_{ij}\mu_i \\
E[\mathrm{Var}[E[k_{ij}|z_{ij},\theta_j,\gamma_j,x_{ij}]|\theta_j,\gamma_j,x_{ij}]] &= \left(\mathrm{Var}[\gamma]+E[\gamma]^2\right) \\
&\quad \alpha_{ij}^2\mu_i\left(E[\theta]-\left(\mathrm{Var}[\theta]+E[\theta]^2\right)\right) \\
E[\mathrm{Var}[E[k_{ij}|\theta_j,\gamma_j,x_{ij}]|\gamma_j,\theta_j]] &= (\mathrm{Var}[\gamma]+E[\gamma]^2)(\mathrm{Var}[\theta]+E[\theta]^2)\alpha_{ij}^2\sigma_i^2 \\
E[\mathrm{Var}[E[k_{ij}|\gamma_j,\theta_j]|\gamma_j]] &= \left(\mathrm{Var}[\gamma]+E[\gamma]^2\right)\mathrm{Var}[\theta]\alpha_{ij}^2\mu_i^2 \\
\mathrm{Var}[E[k_{ij}|\gamma_j]] &= \mathrm{Var}[\gamma]E[\theta]^2\alpha_{ij}^2\mu_i^2
\end{aligned}$$

For each gene, the expectation of the sample variance w_i of the normalized count $\frac{k_{ij}}{\alpha_{ij}}$ is given by

$$\begin{aligned}
E[w_i] \;&=\; \left(\beta_i E[\gamma]E[\theta] + \left(\mathrm{Var}[\gamma]+E[\gamma]^2\right)\left(E[\theta]-\left(\mathrm{Var}[\theta]+E[\theta]^2\right)\right)\right)\mu_i + \\
&\quad \left(\left(\mathrm{Var}[\gamma]+E[\gamma]^2\right)\mathrm{Var}[\theta]+\mathrm{Var}[\gamma]E[\theta]^2\right)\mu_i^2 + \\
&\quad (\mathrm{Var}[\gamma]+E[\gamma]^2)(\mathrm{Var}[\theta]+E[\theta]^2)\sigma_i^2
\end{aligned}$$

The biological variance estimate $\widehat{\sigma_i}^2$ is given by

$$\begin{aligned}\widehat{\sigma_i}^2 \; = \; & \frac{1}{(\widehat{\mathrm{Var}[\gamma]} + \widehat{E[\gamma]}^2)(\widehat{\mathrm{Var}[\theta]} + \widehat{E[\theta]}^2)} \{w_i - \\ & \left(\beta_i \widehat{E[\gamma]}\widehat{E[\theta]} + \left(\widehat{\mathrm{Var}[\gamma]} + \widehat{E[\gamma]}^2\right)\left(\widehat{E[\theta]} - \left(\widehat{\mathrm{Var}[\theta]} + \widehat{E[\theta]}^2\right)\right)\right)\widehat{\mu_i} - \\ & \left(\left(\widehat{\mathrm{Var}[\gamma]} + \widehat{E[\gamma]}^2\right)\widehat{\mathrm{Var}[\theta]} + \widehat{\mathrm{Var}[\gamma]}\widehat{E[\theta]}^2\right)\widehat{\mu_i}^2\right\}\end{aligned}$$

where

$$\widehat{\mu_i} = \frac{k_i}{\widehat{E[\gamma]}\widehat{E[\theta]}}$$

Supplementary Note 4: Adjusting for batch effects

The two single-cell data sets, comprising a set of 80 mESCs cultured in the serum condition and a set of 80 mESCs cultured in the 2i condition, were processed in multiple batches. Two libraries were constructed where the first library contains 40 mESCs (cell 1-40 in serum) and 40 mESC (cell 1-40 in 2i) and the second library contains the remaining cells. The two libraries were split into two halves and sequenced on different lanes, resulting in four batches. For each batch, the same amount of ERCC spike-ins were added. Supplementary Fig. 2a shows a PCA plot of all cells using the number of transcripts of ERCC spike-ins. All cells cluster together according to their batch, indicating that there exists significant non-biological experimental variation across multiple batches. One source of variation within this data set could be the batch-to-batch variability of $E[\gamma]$ and $E[\theta]$. To examine this possibility, we estimated $E[\eta] = E[\gamma]E[\theta]$ and divided k_{ij} by $\widehat{E[\eta]}$ for each batch, which is equivalent to converting the number of sequenced transcripts to the number of transcripts within a single

cell. Supplementary Fig. 2b shows that this normalization procedure adjusts the data for batch effects, suggesting that batch-to-batch variability in sequencing efficiency is the major source of batch effects.

Supplementary Note 5: Validating the model

To validate the model we examine how well it explains the dispersion observed in the real data. The model predicts that the variance of k_{ij} is a quadratic function of the expression level x_i, meaning that the squared CV is inversely proportional to x_i for small values of x_i, but approaches a constant value as the expression level increases. Moreover, the model predicts that the Fano factor is a linear function of x_i. Supplementary Fig. 3 illustrates the fit to the first batch of the mESC data cultured in the 2i condition. The nonlinear least squares estimates of $E[\gamma]$ and $E[\theta]$ are

$$\widehat{E[\gamma]} = 0.2489, \ \widehat{E[\theta]} = 0.0712$$

which suggests that only 1.77% of all transcripts are successfully sequenced in the first batch. In Supplementary Fig. 4 we explore the mean-variance relationship for the two data sets by plotting the squared CV (or the Fano factor) versus the number of added transcripts for all spike-in RNAs (blue circles). The squared CV is inversely proportional to the expression level and the Fano factor remains constant until x_i reaches 100, which is consistent with the model. As the expression level increases, the squared CV approaches a constant value and the Fano factor increases. These observations suggest that the variance is a quadratic function of the expression level, consistent with the predicted mean-variance relationship,

and suggesting that the assumptions underlying the model are valid.

Supplementary Note 6: Simulating single-cell data

We describe two generative models for simulating single-cell data: 1) a "T" model assuming only technical noise and 2) a "T+B" model assuming both technical and biological noise. Given the least squares estimates $\widehat{E[\gamma]}$, $\widehat{E[\theta]}$, $\widehat{\mathrm{Var}[\gamma]}$, $\widehat{\mathrm{Var}[\theta]}$ and $\widehat{\mu_i}$, k_{ij} can be generated according to the following "T" model:

1. The capture efficiency of cell j follows a beta distribution

$$\theta_j \sim \mathrm{Beta}(\theta_j|\alpha_\theta, \beta_\theta)$$

where

$$\begin{aligned}\alpha_\theta &= \widehat{E[\theta]}\left(\frac{\widehat{E[\theta]}(1-\widehat{E[\theta]})}{\widehat{\mathrm{Var}[\theta]}} - 1\right)\\ \beta_\theta &= (1-\widehat{E[\theta]})\left(\frac{\widehat{E[\theta]}(1-\widehat{E[\theta]})}{\widehat{\mathrm{Var}[\theta]}} - 1\right)\end{aligned}$$

It should be noted that the above method-of-moments estimates are only valid if $\widehat{\mathrm{Var}[\theta]} < \widehat{E[\theta]}(1-\widehat{E[\theta]})$.

2. The number of transcripts available for sequencing z_{ij} is selected according to a binomial distribution:

$$z_{ij} \sim \mathrm{Binomial}(z_{ij}|\widehat{\mu_i}, \theta_j)$$

where $\widehat{\mu_i} = \frac{k_i}{\widehat{E[\gamma]}\widehat{E[\theta]}}$ is the estimated number of transcripts of gene i and we do not allow cell-to-cell variability in $\widehat{\mu_i}$.

3. The sequencing efficiency of cell j follows a gamma distribution

$$\gamma_j \sim \text{Gamma}(\gamma_j|\alpha_\gamma, \beta_\gamma)$$

where

$$\begin{aligned} \alpha_\gamma \text{ (shape)} &= \frac{\widehat{E[\gamma]}^2}{\widehat{\text{Var}[\gamma]}} \\ \beta_\gamma \text{ (scale)} &= \frac{\widehat{\text{Var}[\gamma]}}{\widehat{E[\gamma]}} \end{aligned}$$

4. Given z_{ij}, k_{ij} is Poisson distributed such that

$$k_{ij} \sim \text{Poisson}(k_{ij}|\gamma_j \alpha_{ij} z_{ij})$$

The above model can be modified to the "T+B" model by adding the following assumptions

1. The number of transcripts of gene i within cell j (continuous version) follows a gamma distribution

$$x_{ij} \sim \text{Gamma}(x_{ij}|\alpha_{x_i}, \beta_{x_i})$$

where

$$\begin{aligned} \alpha_{x_i} \text{ (shape)} &= \frac{\widehat{\mu_i}^2}{\widehat{\sigma_i}^2} \\ \beta_{x_i} \text{ (scale)} &= \frac{\widehat{\sigma_i}^2}{\widehat{\mu_i}} \end{aligned}$$

where $\widehat{\sigma_i}^2$ is the biological variance estimate. Under the standard two-state kinetic model of stochastic gene expression, a gamma distribution is an approximation of the steady state mRNA density when the rate of gene inactivation is significantly larger than the rate of gene activation and is larger the rate of degradation (short and infrequent bursts of mRNA expression)[4].

2. The number of transcripts available for sequencing z_{ij} is selected according to a binomial distribution:

$$z_{ij} \sim \text{Binomial}(z_{ij}|[x_{ij}], \theta_j)$$

where $[x_{ij}]$ is the round function.

Finally, we generate allele expression counts under two assumptions:

- Unfixed allelic ratio: we separately estimate the mean expression levels of both alleles ($\widehat{\mu_i}^p$ for paternal allele and $\widehat{\mu_i}^m$ for maternal allele) and independently generate k_{ij} for each allele.
- Fixed allelic ratio ($\sigma = 0.5$): we set $\widehat{\mu_i} = (\widehat{\mu_i}^p + \widehat{\mu_i}^m)\sigma$ for the first allele and $\widehat{\mu_i} = (\widehat{\mu_i}^p + \widehat{\mu_i}^m)(1 - \sigma)$ for the second allele and then independently generate k_{ij}.

Supplementary Note 7: Calculating the effective length of SNPs

In the read-based scRNA-seq experiments, the expression level of a gene is partially proportional to the length of the gene. This is also applicable to SNPs since the number of

mappable positions of reads overlapping SNPs differs depending upon the SNPs relative positions within a gene. Given paired-end reads, the number of mappable positions of a SNP (effective length, β) of transcript i, which is used in computing α_{ij}, was calculated by

1. if $l_i < f$, $\beta = 1$.
2. else if $p_i \leq f$ and $p_i \leq r$, $\beta = p_i$.
3. else if $p_i \leq f$ and $p_i > r$, $\beta = r + \max(p_i - f + r, 0)$
4. else, $\beta = 2 * r$.

where f is the length of fragments (200bp in this study), r is the length of reads (100bp), l_i is the length of transcript i, and p_i is the position of the SNP within transcript i. If a SNP is shared by multiple transcript isoforms, we took the maximum of the multiple values of β. Since most intergenic transcripts are adjacent to 5′- and 3′-ends of known genes[5], the effective length of SNPs near the starts and ends of annotated genes would be longer. Consistent with this, we observed that normalizing the raw read counts of these SNPs by their effective length overestimates their expression levels compared to other SNPs (Supplementary Fig. 13). Therefore, we set the effective length of SNPs near the starts and ends of annotated genes ($\beta < r$) to the length of reads r.

Supplementary Note 8: Shared attributes of genes showing stochastic monoallelic expression

To identify the shared features of the 427 genes showing stochastic monoallelic expression (hereafter we define them as the "SM gene set"), we first performed gene ontology (GO) enrichment analysis by setting up the 7,385 genes with one or more expressed SNPs as a background. At a given FDR cutoff (Benjamini-Hochberg adjusted $P < 0.05$), we could not find any significantly enriched GO terms. We next examined whether transcription factor (TF) binding or histone modification (HM) patterns are enriched in the SM gene set. We extracted binary interactions between TF/HM and target genes from the ESCAPE database (61 TFs and 7 HMs), which are experimentally supported by high-throughput studies in mESCs[6]. Since all the genes in the SM gene set are moderately or highly expressed, active marks (DMAP1, E2F1, E2F4, JARID1A, KDM5B, MAX, MED1, MED12, MYC, MYCN, NIPBL, SIN3B, SOX2, ZFP42, H3K4me1, H3K4me2, H3K4me3, H3K36me3, and H3K79me2) are enriched in the SM gene set while inactive marks (EZH2, JARID2, MTF2, RNF2, SUZ12, H3K9me3, and H3K27me3) are depleted (Benjamini-Hochberg adjusted $P <$ 0.05 by Fisher's exact test, background set: 7,385 genes with one or more expressed SNPs). However, when we set up 3,342 genes with ≥ 1 SNP above the expression cutoff (estimated number of transcripts per cell > 2 for both alleles, this cutoff includes all genes showing stochastic monoallelic expression) as a background, no TF/HM pattern is associated with the SM gene set.

Supplementary Note 9: Allelic correlation across cells

We investigated whether the two alleles show independent expression by using Spearman's rank correlation coefficients (ρ) to correlate their normalized read counts across cells. We normalized the raw read counts by size factors estimated from allele counts (Supplementary Note 1). For each SNP, to capture an "L"-shaped pattern with negative correlation between the two alleles (Fig. 3b), we excluded cells with an estimated number of transcripts covering both alleles less than 5. To obtain a null model we independently simulated ASE levels for 54 cells assuming technical noise only or both technical and biological noise (Supplementary Fig. 14a). Under these models, the expression levels of the two alleles should be uncorrelated. However, for both the simulated and real data, we frequently observed an "L"-shaped pattern with negative correlation between the two alleles for lowly or moderately expressed genes. In contrast, for highly expressed genes, the observed correlation coefficients were positive, while simulated correlation coefficients from the two models were ~ 0. As expected, when we calculated the Spearman's ρ without excluding cells expressing two alleles very lowly, we observed no correlations from both models at all expression levels (Supplementary Fig. 14b). More specifically, of the 2,685 genes with ≥ 1 SNP above the expression cutoff (estimated number of transcripts per cell > 1 for both alleles), 66 had significantly higher correlation than expected (empirical Benjamini-Hochberg adjusted $P < 0.05$ from 10,000 simulated samples). Since these genes are highly expressed in general, genes involved in protein synthesis (e.g. "ribosome") were enriched (Benjamini-Hochberg adjusted $P < 0.05$, we set up the 2,685 genes as a background).

Supplementary References

1. Grün, D., Kester, L. & van Oudenaarden, A. Validation of noise models for single-cell transcriptomics. *Nature Methods* **11**, 637–640 (2014).

2. Anders, S. & Huber, W. Differential expression analysis for sequence count data. *Genome Biology* **11**, R106 (2010).

3. Bowsher, C. G. & Swain, P. S. Identifying sources of variation and the flow of information in biochemical networks. *Proceedings of the National Academy of Sciences, USA* **109**, E1320–E1329 (2012).

4. Raj, A., Peskin, C. S., Tranchin, D., Vargas, D. Y. & Tyagi, S. Stochastic mRNA synthesis in mammalian cells. *PLoS Biology* **4**, e309 (2006).

5. van Bakel, H., Nislow, C., Blencowe, B. J. & Hughes, T. R. Most "dark matter" transcripts are associated with known genes. *PLoS Biology* **8**, e1000371 (2010).

6. Xu, H., Ang, Y. S., Sevilla, A., Lemischka, I. R. & Ma'ayan, A. Construction and validation of a regulatory network for pluripotency and self-renewal of mouse embryonic stem cells. *PLoS Computational Biology* **10**, e1003777 (2014).
