# Supplementary material for: Characterizing noise structure in single-cell RNA-seq distinguishes genuine from technical stochastic allelic expression
Source: Nat Commun. 2015 Oct 22;6:8687. doi: 10.1038/ncomms9687 (PMC4627577; doi:10.1038/ncomms9687)
Supplement: Supplementary Software — Ancillary files and scripts that are necessary to run the code. [file ncomms9687-s3.zip › SupplementaryCode/supplementarySoftwareGuide.pdf]

Supplementary Information II: Characterizing noise structure in single-cell RNA-seq distinguishes genuine from technical allelic expression

Jong Kyoung Kim

Monday, May 25, 2015

In this document, we show the complete workflow to estimate the biological variance and its related noise measure of a gene, and to simulate single-cell data under various assumptions from single-cell RNA-seq (scRNA-seq) data.

1 Preparations

1.1 Packages

We load all R and Bioconductor packages we need in this analysis.

```
library(plyr)
library(minpack.lm)
library(ggplot2)
library(DESeq)
```

1.2 Functions

We load all R functions in noise_decomposition_function.R, which is available in the SupplementaryCode.zip. You should first unzip the zip file and set your working directory by selecting the extracted folder.

```
source("noise_decomposition_function.R")
```

1.3 Input files

All input files used in this analysis are available in the SupplementaryCode.zip.

2 Estimating biological noise from UMI-based scRNA-seq

In this section, we show how to conduct a noise decomposition analysis in UMI-based scRNA-seq data of Grün *et al.* (2014).

2.1 Preparing the count table

We load the estimated number of spiked-in mRNA molecules of the 92 ERCC spike-ins from ercc_counts.txt.

```r
erccNumber = read.table("ercc_counts.txt", stringsAsFactors = FALSE, header=TRUE)
rownames(erccNumber) = erccNumber[,1]
erccSeqID = sort(rownames(erccNumber))
head(erccNumber)
```

```
##            Re.sort.ID nb.cell.mix.1.500000
## ERCC-00130 ERCC-00130            10839.855
## ERCC-00002 ERCC-00002             5419.928
## ERCC-00074 ERCC-00074             5419.928
## ERCC-00096 ERCC-00096             5419.928
## ERCC-00004 ERCC-00004             2709.964
## ERCC-00046 ERCC-00046             1354.982
```

We next load the number of sequenced mRNA molecules of the spike-ins for each cell from ercc_cell_counts.txt. Notice that the numbers in ercc_cell_counts.txt correspond to the number of mRNA molecules that is transformed from the raw UMI count to account for the stochastic labeling process of the UMI protocol.

```r
erccCount = read.table("ercc_cell_counts.txt", stringsAsFactors = FALSE, header=TRUE)
rownames(erccCount) = erccCount[,1]
erccCount = erccCount[,-1]
head(erccCount[1:5, 1:6])
```

```
##               SC_2i_1    SC_2i_2    SC_2i_3   SC_2i_4   SC_2i_5    SC_2i_6
## ERCC-00002 152.682894 121.925950 161.925775 156.34010 93.028837 113.999620
## ERCC-00003   8.127667  10.200553  13.341696  13.34170  8.127667   3.017717
## ERCC-00004  67.065271  33.043723  59.384695  56.87487 36.480016  45.875566
## ERCC-00009   3.017717   6.071431   7.097484  10.20055  4.031579   6.071431
## ERCC-00014   0.000000   0.000000   0.000000   0.00000  0.000000   0.000000
```

Finally, we load the number of sequenced mRNA molecules of genes for each cell from gene_cell_counts.txt.

```r
countGenes = read.table("gene_cell_counts.txt", stringsAsFactors = FALSE, header=TRUE)
```

We replace one of the duplicated gene symbol ("Mar-02" not a gene symbol but an annotation error in the original file) to "Mar-02-1".

```r
countGenes[6223,1] = "Mar-02-1"
nameGenes = countGenes[,1]
countGenes = countGenes[,-1]
rownames(countGenes) = nameGenes
```

We also discard cells with fewer than 500 sequenced transcripts for ERCC spike-ins and 10,000 sequenced transcripts for endogenous genes.

```r
removeCells = colSums(erccCount)>500 & colSums(countGenes)>10000
erccCount = erccCount[, removeCells]
countGenes = countGenes[, removeCells]
head(countGenes[1:5, 1:6])
```

```
##                 SC_2i_1  SC_2i_2  SC_2i_3  SC_2i_4  SC_2i_5  SC_2i_6
## 0610007N19Rik 0.000000 0.000000 0.000000 0.000000 0.000000 0.000000
```

```
## 0610007P14Rik 3.017717 0.000000 2.007853 6.071431 0.000000 2.007853
## 0610009B22Rik 1.001958 0.000000 0.000000 0.000000 0.000000 2.007853
## 0610009D07Rik 3.017717 1.001958 4.031579 5.049473 3.017717 4.031579
## 0610009O20Rik 0.000000 0.000000 5.049473 4.031579 5.049473 0.000000
```

2.2 Adjusting for batch effects

The 80 2i-grown mESCs were processed in two batches. Two libraries were constructed where the first library contains 40 mESCs (cell 1-40) and the second library contains the remaining cells (cell 41-80). To adjust for batch effects, we normalize the raw number of sequenced transcripts by dividing the numbers by $E[\gamma]E[\theta]$, separately for each batch.

```
cellCondition = "SC_2i"
nCount = selectCells(erccCount, countGenes, cellCondition, 1, 40, erccSeqID, erccNumber)
nCountSpikes = nCount[[1]]
numberSpikes = nCount[[2]]
nCountGenes = nCount[[3]]
sizeFactorMatrix = matrix(1, nrow=nrow(nCountSpikes), ncol=ncol(nCountSpikes))
gammaThetaEstimate = estimateGammaTheta(nCountSpikes, numberSpikes, sizeFactorMatrix)
EGammaThetaSC2i1 = gammaThetaEstimate$gammaTheta[[1]]
nCountGenesSC2i1 = nCountGenes / EGammaThetaSC2i1
nCountSpikesSC2i1 = nCountSpikes / EGammaThetaSC2i1
cellCondition = "SC_2i"
nCount = selectCells(erccCount, countGenes, cellCondition, 41, 80, erccSeqID, erccNumber)
nCountSpikes = nCount[[1]]
numberSpikes = nCount[[2]]
nCountGenes = nCount[[3]]
sizeFactorMatrix = matrix(1, nrow=nrow(nCountSpikes), ncol=ncol(nCountSpikes))
gammaThetaEstimate = estimateGammaTheta(nCountSpikes, numberSpikes, sizeFactorMatrix)
EGammaThetaSC2i2 = gammaThetaEstimate$gammaTheta[[1]]
nCountGenesSC2i2 = nCountGenes / EGammaThetaSC2i2
nCountSpikesSC2i2 = nCountSpikes / EGammaThetaSC2i2
```

2.3 Quantifying biological noise

The biological variance and its related noise measures can be estimated using a function, estimateBiological-Variance. It returns a data frame, noiseEstimateSC2i, with mean estimates $\hat{\mu_i}$, biological variance estimates $\hat{\sigma_i}^2$, biological squared coefficient of variation estimates, biological Fano factor estimates, and the fraction of biological variance. Notice that some values of the biological variance estimate in the data frame can be set to zero for either one of the following reasons:

1. If all cells of a gene have zero counts, the biological varianc estimate will be set to zero, and the other noise measures will be set to `NA`.
2. If the expected technical variance of a gene exceeds the sample variance, all variance related estimates will be set to zero.

```
nCountSpikesSC2i = cbind(nCountSpikesSC2i1, nCountSpikesSC2i2)
nCountGenesSC2i = cbind(nCountGenesSC2i1, nCountGenesSC2i2)
sizeFactorMatrixSC2i = matrix(1, nrow=nrow(nCountSpikesSC2i),
                               ncol=ncol(nCountSpikesSC2i))
sizeFactorMatrixSC2iGenes = matrix(1, nrow=nrow(nCountGenesSC2i),
```

```
                                     ncol=ncol(nCountGenesSC2i))
noiseEstimateSC2i = estimateBiologicalVariance(nCountGenesSC2i, nCountSpikesSC2i,
                                               sizeFactorMatrixSC2i, numberSpikes,
                                               sizeFactorMatrixSC2iGenes)
head(noiseEstimateSC2i)
```

```
##                predictedCount predictedBVariance predictedCV2 predictedFano
## 0610007N19Rik         0.00000              0.000          NaN           NaN
## 0610007P14Rik       118.04115           2883.129    0.2069175      24.42478
## 0610009B22Rik        31.18636              0.000    0.0000000       0.00000
## 0610009D07Rik       195.24146          11277.758    0.2958548      57.76313
## 0610009O20Rik        33.07284           2090.795    1.9114746      63.21789
## 0610010B08Rik       318.27866          11953.477    0.1179992      37.55664
##                fracBVariance
## 0610007N19Rik            NaN
## 0610007P14Rik      0.3220891
## 0610009B22Rik      0.0000000
## 0610009D07Rik      0.5171019
## 0610009O20Rik      0.5652750
## 0610010B08Rik      0.3931034
```

2.4 Technical noise fit

We first estimate the four parameters $(E[\gamma], \text{Var}[\gamma], E[\theta], \text{Var}[\theta])$ capturing technical variability from the spike-ins .

```
EVGammaThetaEstimate = estimateEVGammaTheta(nCountSpikesSC2i,
                                            numberSpikes, sizeFactorMatrixSC2i)
EGamma = EVGammaThetaEstimate$EGamma
ETheta = EVGammaThetaEstimate$ETheta
E2Gamma = EVGammaThetaEstimate$E2Gamma
E2Theta = EVGammaThetaEstimate$E2Theta
VGamma = EVGammaThetaEstimate$VGamma
VTheta = EVGammaThetaEstimate$VTheta
```

We then estimate the exptected technical variance $\text{Var}[k_{ij}]$. The first figure is for Figure S5a (Squared CV is plotted against the estimated number of transcripts per cell) and the second for Figure S5b (Fano factor is plotted against the estimated number of transcripts per cell).

```
par(cex.axis=1, cex.lab=1)
plot( NULL, xaxt="n",
      log="xy", xlim = c( 1e-2, 1e5 ), ylim = c(0.01, 100),
      xlab = "Estimated number of transcripts per cell", ylab = "Squared CV" )
axis( 1, 10^(-2:5), c("0.01", "0.1", "1", "10", "100", "1000",
                      expression(10^4), expression(10^5)) )
points(rowMeans(nCountGenesSC2i) / (EGamma*ETheta),
       apply(nCountGenesSC2i, 1, var)/rowMeans(nCountGenesSC2i)^2,
       pch=20, cex=0.7, col="darkgray")
points(numberSpikes[,1], apply(nCountSpikesSC2i, 1, var)/rowMeans(nCountSpikesSC2i)^2,
       pch=20, cex=0.7, col="darkblue")
xg = 10^seq(-2, 5, length.out=1000)
predictedVariance = EGamma*ETheta*xg + (VGamma+E2Gamma)*(ETheta-(E2Theta+VTheta))*xg +
```

```
  (VGamma+E2Gamma)*VTheta*xg^2 + (E2Theta*VGamma)*xg^2
lines(xg, predictedVariance/(xg*EGamma*ETheta )^2, col="indianred", lwd=3)
```

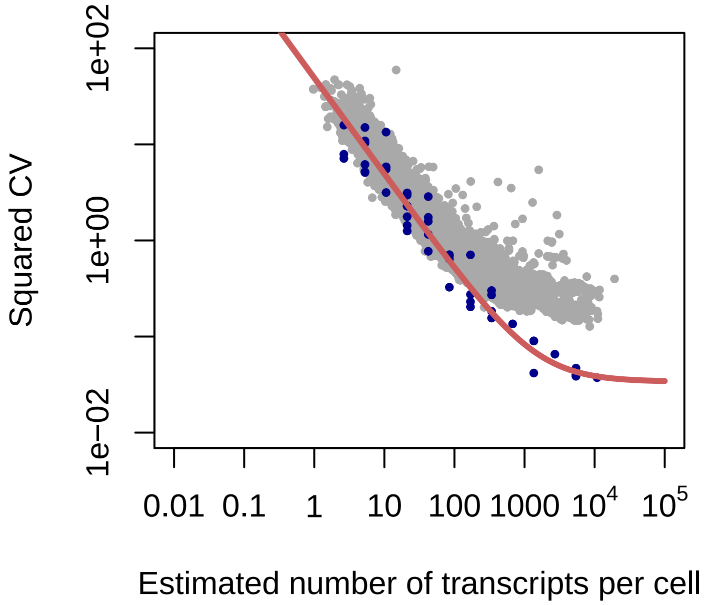


```
par(cex.axis=1, cex.lab=1)
plot( NULL, xaxt="n",
      log="xy", xlim = c( 1e-2, 1e5 ), ylim = c(1, 10000),
      xlab = "Estimated number of transcripts per cell", ylab = "Fano factor" )
axis( 1, 10^(-2:5), c("0.01", "0.1", "1", "10", "100", "1000",
                      expression(10^4), expression(10^5)) )
points(rowMeans(nCountGenesSC2i) / (EGamma*ETheta),
       apply(nCountGenesSC2i, 1, var)/rowMeans(nCountGenesSC2i),
       pch=20, cex=0.7, col="darkgray")
points(numberSpikes[,1], apply(nCountSpikesSC2i, 1, var)/rowMeans(nCountSpikesSC2i),
       pch=20, cex=0.7, col="darkblue")
xg = 10^seq(-2, 5, length.out=1000)
predictedVariance = EGamma*ETheta*xg + (VGamma+E2Gamma)*(ETheta-(E2Theta+VTheta))*xg +
  (VGamma+E2Gamma)*VTheta*xg^2 + (E2Theta*VGamma)*xg^2
lines(xg, predictedVariance/(xg*EGamma*ETheta ), col="indianred", lwd=3)
```

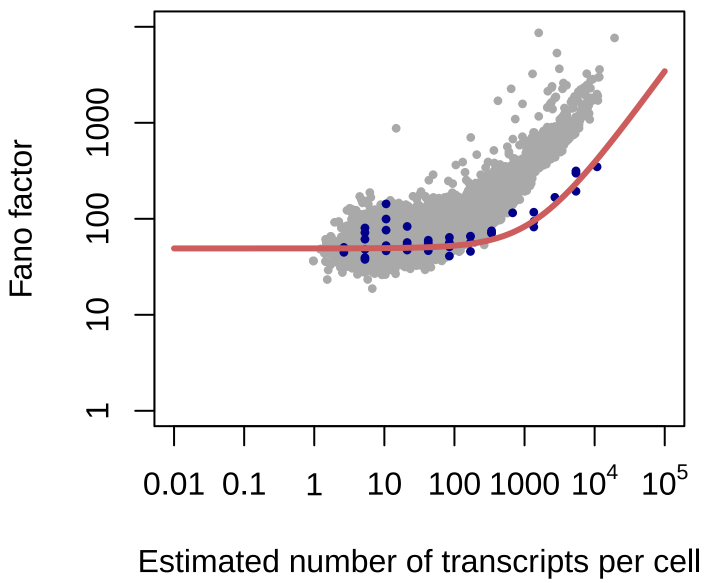

2.5 Simulating single-cell data

Our model allows single-cell data to be simulated from the generative model. To simulate single-cell data under the T model (assuming only technical noise), we simply set the biological variance term to zero.

```
meanMoleculesGenes2i = noiseEstimateSC2i$predictedCount
simulatedCountSpikes2i = simulateCountGenes(meanMoleculesGenes2i, ETheta, VTheta,
                                            EGamma, VGamma, sizeFactorMatrixSC2iGenes,
                                            ncol(nCountGenesSC2i), 0*noiseEstimateSC2i[,2])
```

If biological variability is allowed, we can simulate single-cell data under the T+B model (assuming both technical and biological noise).

```
simulatedCountGenes2i = simulateCountGenes(meanMoleculesGenes2i, ETheta, VTheta,
                                           EGamma, VGamma, sizeFactorMatrixSC2iGenes,
                                           ncol(nCountGenesSC2i), noiseEstimateSC2i[,2])
```

The following figure will be similar to Figure S10a. Squared CV of simulated genes in 2i-grown mESCs using the UMI protocol are plotted against the estimated number of transcripts per cell.

```
par(mar=c(5,5,1,1), cex.axis=1, cex.lab=1)
plot( NULL, xaxt="n",
      log="xy", xlim = c( 1e-2, 1e5 ), ylim = c(1e-4, 100),
      xlab = "Estimated number of transcripts per cell", ylab = "Squared CV" )
axis( 1, 10^(-2:5), c("0.01", "0.1", "1", "10", "100", "1000",
                      expression(10^4), expression(10^5)) )
points(rowMeans(simulatedCountGenes2i) / (EGamma*ETheta),
```

```
       apply(simulatedCountGenes2i, 1, var)/rowMeans(simulatedCountGenes2i)^2,
       pch=20, cex=0.7, col="darkgray")
points(rowMeans(simulatedCountSpikes2i) / (EGamma*ETheta),
       apply(simulatedCountSpikes2i, 1, var)/rowMeans(simulatedCountSpikes2i)^2,
       pch=20, cex=0.7, col="darkblue")
xg <- 10^seq(-2, 5, length.out=1000)
predictedVariance = EGamma*ETheta*xg + (VGamma+E2Gamma)*(ETheta-(E2Theta+VTheta))*xg +
  (VGamma+E2Gamma)*VTheta*xg^2 + (E2Theta*VGamma)*xg^2
lines(xg, predictedVariance/(xg*EGamma*ETheta )^2, col="indianred", lwd=3)
legend(0.01, 0.01, legend=c("Genes with T+B", "Genes with T",
                            "Technical noise fit by ERCC spike-ins"),
       pch=c(20, 20, NA), lty=c(NA, NA, 1), lwd=c(NA, NA, 3),
       col=c("darkgray", "darkblue", "indianred"), bty="n", cex=0.7)
```

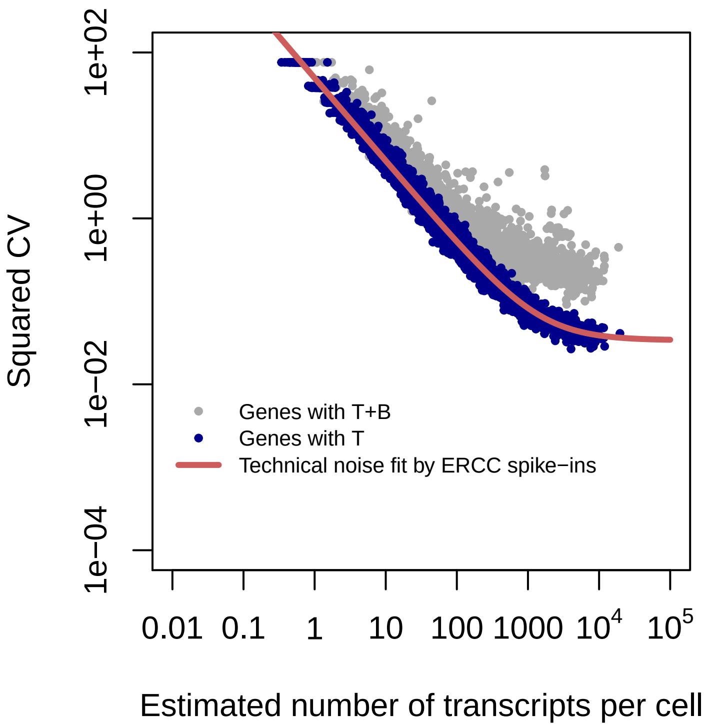

3 Estimating biological noise from read-based scRNA-seq

In this section, we show how to conduct a noise decomposition analysis and how to simulated single-cell data under variation assumptions in read-based scRNA-seq data.

3.1 Preparing the count table

We load the estimated number of spiked-in mRNA molecules of the 92 ERCC spike-ins from mESC_2i_ERCC92TrueNumber.txt. Notice that we always assume the ERCC spike-ins are sorted in ascending order of their identifiers.

```r
numberSpikes = read.table("mESC_2i_ERCC92TrueNumber.txt",
                          header=TRUE, sep="\t", stringsAsFactors = FALSE,
                          row.names=1, check.names = FALSE)
head(numberSpikes)
```

```
##            concentration.in.Mix.1..attomoles.ul.
## ERCC-00002                          4.064945e+04
## ERCC-00003                          2.540591e+03
## ERCC-00004                          2.032473e+04
## ERCC-00009                          2.540591e+03
## ERCC-00012                          3.101307e-01
## ERCC-00013                          2.481046e+00
##            concentration.in.Mix.2..attomoles.ul.
## ERCC-00002                          8.129891e+04
## ERCC-00003                          5.081182e+03
## ERCC-00004                          5.081182e+03
## ERCC-00009                          2.540591e+03
## ERCC-00012                          4.651961e-01
## ERCC-00013                          4.962092e+00
```

We then load the raw read counts of the spike-ins for each cell from mESC_2i_ERCC92.txt.

```r
countSpikes = read.table("mESC_2i_ERCC92.txt",
                         header=TRUE, sep="\t", stringsAsFactors = FALSE,
                         row.names=1, check.names = FALSE)
head(countSpikes[1:5, 1:6])
```

```
##                 1      2      3      4      5     6
## ERCC-00002 951584 540833 502971 431392 232247 98741
## ERCC-00003  53939  25385  34790  26986  13910  5221
## ERCC-00004 207290 131923 134711 107336  57388 28204
## ERCC-00009  95644  76894  62968  66314  29212  8843
## ERCC-00012      0      0      0      0      0    39
```

We exclude bad quality cells from 96 cells according to our QC criteria (see "Quality control on cells" in the Methods).

```r
badIndex = c(1,  3,  9, 14, 22, 24, 27, 29, 32, 34, 35,
             39, 40, 44, 48, 50, 51, 53, 54, 55, 57, 59,
             60, 61, 63, 64, 67, 68, 69, 70, 71, 72, 74,
             75, 81, 85, 86, 89, 93, 94, 95, 96)
countSpikes = countSpikes[,setdiff(1:96, badIndex)]
numGene <- nrow(countSpikes)
numCell <- ncol(countSpikes)
```

Finally, we load the annotation of each SNP and the raw read counts of the two alleles (bad quality cells have been already removed).

```r
snpAnnot = read.table("mESC_2i_snpAnnot.txt", header=TRUE,
                      sep="\t", stringsAsFactors = FALSE,
                      row.names=1, check.names = FALSE)
```

```
countAlleleBL6 = read.table("mESC_2i_BL6Count.txt", header=TRUE,
                            sep="\t", stringsAsFactors = FALSE,
                            row.names=1, check.names = FALSE)
countAllele129 = read.table("mESC_2i_129Count.txt", header=TRUE,
                            sep="\t", stringsAsFactors = FALSE,
                            row.names=1, check.names = FALSE)
```

3.2 Normalization

We calculate two DESeq size factors for each cell, where one is from the ERCC spike-ins (technical size factor capturing cell-to-cell variability in sequencing depth) and another is from endogenous genes (biological size factor capturing cell-to-cell variability in both sequencing depth and the total amount of mRNA molecules).

3.2.1 Normalization for spike-ins

We estimate the transcript length of the spike-ins from ERCC92.fa.

```
library(ShortRead)
erccSeq = readFasta("ERCC92.fa")
erccSeqID = ShortRead::id(erccSeq)
erccLength = width(erccSeq)
names(erccLength) = erccSeqID
detach("package:ShortRead", unload=TRUE)
head(erccLength)
```

```
## ERCC-00002 ERCC-00003 ERCC-00004 ERCC-00009 ERCC-00012 ERCC-00013
##       1061       1023        523        984        994        808
```

In this study, the length of reads and the length of fragments are to 100bp and 200bp, respectively. See Supplementary Note 7 for more details.

```
readLength = 100
fragmentLength = 200
```

We normalize the raw read counts of the spike-ins based on the technical size factors and the effective length.

```
sizeFactor = estimateSizeFactorsForMatrix(countSpikes)
sizeFactorMatrix = (repmat(t(as.matrix(sizeFactor)), numGene, 1) *
                       repmat(as.matrix( (erccLength[rownames(countSpikes)]
                                          -fragmentLength+1)/10^3), 1, numCell))
nCountSpikes = as.matrix(countSpikes)/sizeFactorMatrix
meanSpikes = rowMeans(nCountSpikes)
```

3.2.1 Normalization for endogeneous genes

We load the effective length of SNPs from effectiveSNPLength.RData.

```
load("effectiveSNPLength.RData")
# If effectiveSNPLength.RData is not available, run the following code
#
# effectiveSNPLength = estimateSNPLength(snpAnnot, fragmentLength, readLength)
# effectiveSNPLength = aaply(1:length(effectiveSNPLength), 1, function(x) {
#   max(effectiveSNPLength[snpAnnot[,8]==snpAnnot[x,8] & snpAnnot[,7]==snpAnnot[x,7]])
# }, .progress="text", .expand=FALSE)
# effectiveSNPLength[effectiveSNPLength<readLength] = readLength
```

We then normalize the raw read counts of the two alleles (and the sum of them) based on the biological size factors and the effective length.

```
sizeFactor = estimateSizeFactorsForMatrix(cbind(countAlleleBL6+countAllele129))
sizeFactorMatrixAlleles = (repmat(t(as.matrix(sizeFactor)), nrow(countAlleleBL6), 1) *
                             repmat(as.matrix(effectiveSNPLength/10^3), 1,
                                    ncol(countAlleleBL6)))
nCountAlleles = as.matrix(cbind(countAlleleBL6+countAllele129))/sizeFactorMatrixAlleles
nCountAllele129 = as.matrix(countAllele129)/sizeFactorMatrixAlleles
nCountAlleleBL6 = as.matrix(countAlleleBL6)/sizeFactorMatrixAlleles
```

3.3 Quantifying biological noise

We separately estimate the biological noise of the maternal and paternal alleles.

```
noiseEstimate129 = estimateBiologicalVariance(nCountAllele129, nCountSpikes,
                                              sizeFactorMatrix, numberSpikes,
                                              sizeFactorMatrixAlleles)
noiseEstimateBL6 = estimateBiologicalVariance(nCountAlleleBL6, nCountSpikes,
                                              sizeFactorMatrix, numberSpikes,
                                              sizeFactorMatrixAlleles)
```

3.4 Technical noise fit

We estimate the four parameters capturing technical variability from the ERCC spike-ins.

```
EVGammaThetaEstimate = estimateEVGammaTheta(nCountSpikes, numberSpikes, sizeFactorMatrix)
EGamma = EVGammaThetaEstimate$EGamma
ETheta = EVGammaThetaEstimate$ETheta
E2Gamma = EVGammaThetaEstimate$E2Gamma
E2Theta = EVGammaThetaEstimate$E2Theta
VGamma = EVGammaThetaEstimate$VGamma
VTheta = EVGammaThetaEstimate$VTheta
```

The first figure is for Figure S8a (paternal alleles) and the second for Figure S8c (maternal alleles).

```
par(cex.axis=1, cex.lab=1)
plot( NULL, xaxt="n",
      log="xy", xlim = c( 1e-3, 1e6 ), ylim = c(1e-4, 100),
      xlab = "Estimated number of transcripts per cell", ylab = "Squared CV" )
axis( 1, 10^(-3:6), c("0.001", "0.01", "0.1", "1", "10", "100", "1000",
```

```
                            expression(10^4), expression(10^5), expression(10^6)) )
points(noiseEstimateBL6$predictedCount,
       apply(nCountAlleleBL6, 1, var)/rowMeans(nCountAlleleBL6)^2,
       pch=20, cex=0.7, col="darkgray")
points(numberSpikes[,1],
       apply(nCountSpikes, 1, var)/rowMeans(nCountSpikes)^2,
       pch=20, cex=0.7, col="darkblue")
xg <- 10^seq(-3, 6, length.out=1000)
predictedVariance = EGamma*ETheta*xg / mean(sizeFactorMatrix) +
  (VGamma+E2Gamma)*(ETheta-(E2Theta+VTheta))*xg +
  (VGamma+E2Gamma)*VTheta*xg^2 + (E2Theta*VGamma)*xg^2
lines(xg, predictedVariance/(xg*EGamma*ETheta *mean(sizeFactorMatrix))^2,
      col="indianred", lwd=3)
```

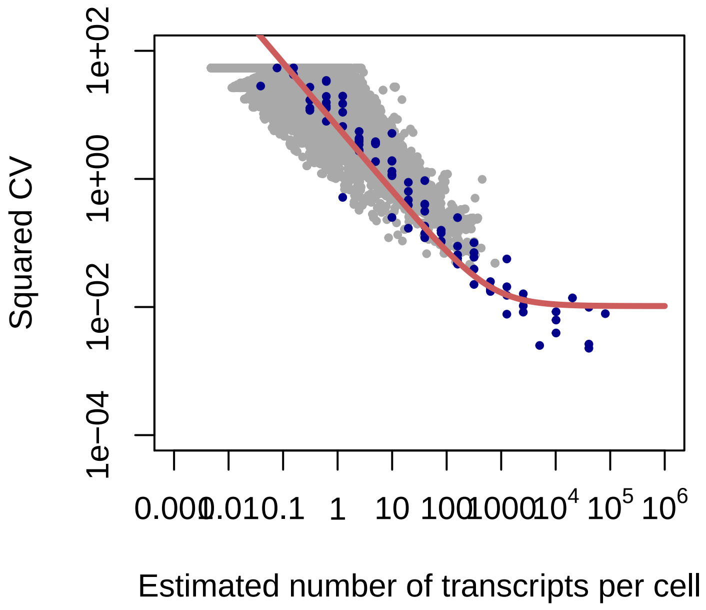


```
par(cex.axis=1, cex.lab=1)
plot( NULL, xaxt="n",
      log="xy", xlim = c( 1e-3, 1e6 ), ylim = c(1e-4, 100),
      xlab = "Estimated number of transcripts per cell", ylab = "Squared CV" )
axis( 1, 10^(-3:6), c("0.001", "0.01", "0.1", "1", "10", "100", "1000",
                            expression(10^4), expression(10^5), expression(10^6)) )
points(noiseEstimate129$predictedCount,
       apply(nCountAllele129, 1, var)/rowMeans(nCountAllele129)^2,
       pch=20, cex=0.7, col="darkgray")
points(numberSpikes[,1],
       apply(nCountSpikes, 1, var)/rowMeans(nCountSpikes)^2,
       pch=20, cex=0.7, col="darkblue")
xg <- 10^seq(-3, 6, length.out=1000)
predictedVariance = EGamma*ETheta*xg / mean(sizeFactorMatrix) +
```

```
  (VGamma+E2Gamma)*(ETheta-(E2Theta+VTheta))*xg +
  (VGamma+E2Gamma)*VTheta*xg^2 + (E2Theta*VGamma)*xg^2
lines(xg, predictedVariance/(xg*EGamma*ETheta *mean(sizeFactorMatrix))^2,
      col="indianred", lwd=3)
```

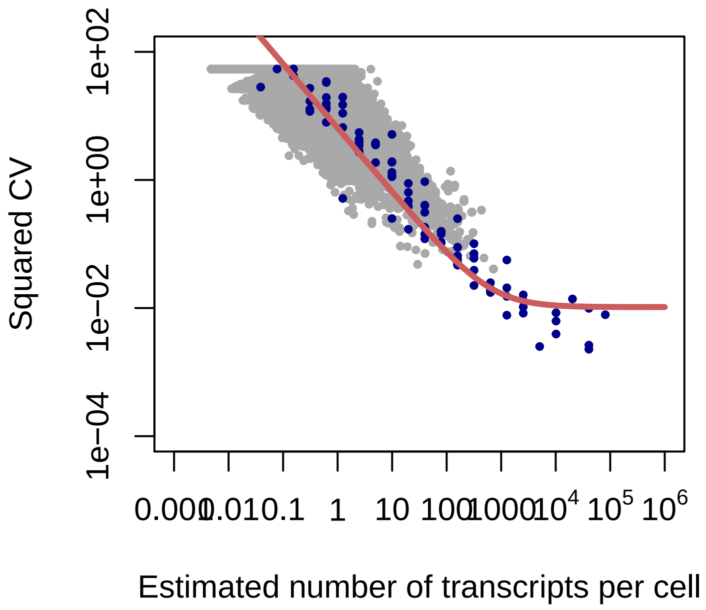

3.5 Simulating single-cell data

Allele-specific single-cell data can be simulated under the T and T+B models.

```
meanMoleculesAllele129 = noiseEstimate129$predictedCount
simulatedCountAllele129 = simulateCountGenes(meanMoleculesAllele129,
                                             ETheta, VTheta, EGamma, VGamma,
                                             sizeFactorMatrixAlleles,
                                           ncol(nCountAllele129), noiseEstimate129[,2])
simulatedCountSpikes129 = simulateCountGenes(meanMoleculesAllele129,
                                             ETheta, VTheta, EGamma, VGamma,
                                             sizeFactorMatrixAlleles,
                                            ncol(nCountAllele129), 0*noiseEstimate129[,2])
nSimulatedCountAllele129 = simulatedCountAllele129/sizeFactorMatrixAlleles
nSimulatedCountSpikes129 = simulatedCountSpikes129/sizeFactorMatrixAlleles
```

The following figure will be similar to Figure S10b. Squared CV of simulated maternal alleles in 2i-grown mESCs using the full length protocol are plotted against the estimated number of transcripts per cell.

```
par(mar=c(5,5,1,1), cex.axis=1, cex.lab=1)
plot( NULL, xaxt="n",
```

```
      log="xy", xlim = c( 1e-2, 1e5 ), ylim = c(1e-4, 100),
      xlab = "Estimated number of transcripts per cell", ylab = "Squared CV" )
axis( 1, 10^(-2:5), c("0.01", "0.1", "1", "10", "100", "1000",
                      expression(10^4), expression(10^5)) )
points(rowMeans(nSimulatedCountAllele129) / (EGamma*ETheta),
       apply(nSimulatedCountAllele129, 1, var)/rowMeans(nSimulatedCountAllele129)^2,
       pch=20, cex=0.7, col="darkgray")
points(rowMeans(nSimulatedCountSpikes129) / (EGamma*ETheta),
       apply(nSimulatedCountSpikes129, 1, var)/rowMeans(nSimulatedCountSpikes129)^2,
       pch=20, cex=0.7, col="darkblue")
xg <- 10^seq(-2, 5, length.out=1000)
predictedVariance = EGamma*ETheta*xg / mean(sizeFactorMatrixAlleles) +
  (VGamma+E2Gamma)*(ETheta-(E2Theta+VTheta))*xg +
  (VGamma+E2Gamma)*VTheta*xg^2 + (E2Theta*VGamma)*xg^2
lines(xg, predictedVariance/(xg*EGamma*ETheta)^2, col="indianred", lwd=3)
legend(0.01, 0.01, legend=c("Alleles with T+B", "Alleles with T",
                            "Technical noise fit by ERCC spike-ins"),
       pch=c(20, 20, NA), lty=c(NA, NA, 1), lwd=c(NA, NA, 3),
       col=c("darkgray", "darkblue", "indianred"), bty="n", cex=0.7)
```

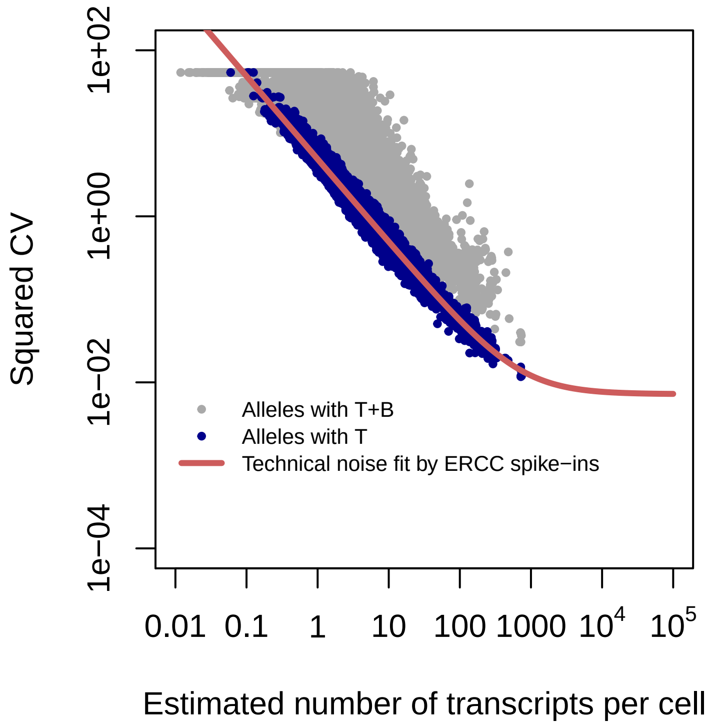

3.5 Testing stochastic allele-specific expression

As a final application, we show how to conduct a null-hypothesis significance testing to determine whether a gene displays a genuine stochastc allele-specific expression.

We first compute the allelic ratio and its average across cells for each allele.

```
meanExpression = noiseEstimateBL6[,1]+noiseEstimate129[,1]
allelicRatio = pmax(as.matrix(countAlleleBL6), as.matrix(countAllele129), na.rm=TRUE) /
(countAlleleBL6+countAllele129)
meanAllelicRatio = rowMeans(allelicRatio, na.rm=TRUE)
```

We choose an SNP assiged to Trim25 as an example.

```
gindex = 181
```

A scatter plot shows the expression values of single cellc, where the x axis represents the normalized read count of the paternal allele and the y axis represents the normalized read count of the maternal allele of the same gene.

```
par(mar=c(5,5,1,1), cex.axis=1, cex.lab=1)
plot(nCountAlleleBL6[gindex,], nCountAllele129[gindex,], pch=20,
     xlab="Normalized read count of paternal allele",
     ylab="Normalized read count of maternal allele",
     xlim=c(0,800), ylim=c(0,800), title="Trim25")
title(main="Trim25", font=1)
```

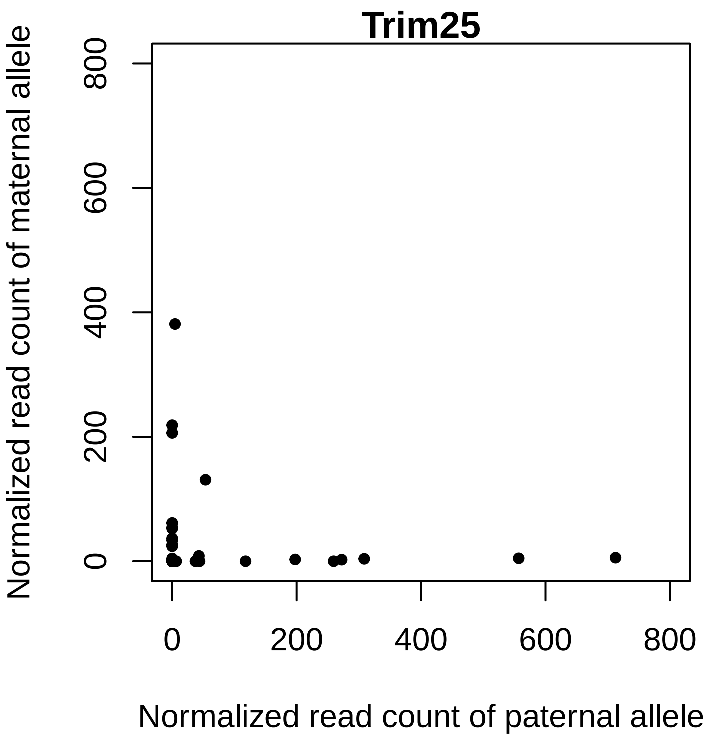

Since the empirical P-value is less than 0.01, we reject the null hypothesis that the observed mean allelic ratio can be explained by technical noise, indicatingi that Trim25 displays a genuine stochastic allele-specific expression.

```
nSample = 10000
count1 = simulateCountEfficient(noiseEstimateBL6[gindex,1], ETheta, VTheta, EGamma, VGamma,
                                sizeFactorMatrixAlleles[gindex,], numCell, 0, nSample)
```

```r
count2 = simulateCountEfficient(noiseEstimate129[gindex,1], ETheta, VTheta, EGamma, VGamma,
                                sizeFactorMatrixAlleles[gindex,], numCell, 0, nSample)
simulated = rowMeans( pmax(count1, count2) / (count1 + count2), na.rm=TRUE)
Pvalue = (sum(simulated>=meanAllelicRatio[gindex], na.rm=T) + 1) /
  (sum(!is.na(simulated))+1)
cat("Empirical P-value = ", Pvalue, "\n")
```

```
## Empirical P-value =  0.00049995
```

4 Session Info

```r
sessionInfo()
```

```
## R version 3.1.2 (2014-10-31)
## Platform: x86_64-w64-mingw32/x64 (64-bit)
##
## locale:
## [1] LC_COLLATE=English_United Kingdom.1252
## [2] LC_CTYPE=English_United Kingdom.1252
## [3] LC_MONETARY=English_United Kingdom.1252
## [4] LC_NUMERIC=C
## [5] LC_TIME=English_United Kingdom.1252
##
## attached base packages:
## [1] stats4    parallel  stats     graphics  grDevices utils     datasets
## [8] methods   base
##
## other attached packages:
##  [1] GenomicAlignments_1.2.1 Rsamtools_1.18.2
##  [3] GenomicRanges_1.18.4    GenomeInfoDb_1.2.4
##  [5] Biostrings_2.34.1       XVector_0.6.0
##  [7] IRanges_2.0.1           S4Vectors_0.4.0
##  [9] BiocParallel_1.0.3      knitr_1.10.5
## [11] DESeq_1.18.0            lattice_0.20-29
## [13] locfit_1.5-9.1          Biobase_2.26.0
## [15] BiocGenerics_0.12.1     ggplot2_1.0.0
## [17] minpack.lm_1.1-8        plyr_1.8.1
##
## loaded via a namespace (and not attached):
##  [1] annotate_1.44.0      AnnotationDbi_1.28.1 base64enc_0.1-2
##  [4] BatchJobs_1.5        BBmisc_1.9           bitops_1.0-6
##  [7] brew_1.0-6           checkmate_1.5.1      codetools_0.2-10
## [10] colorspace_1.2-4     DBI_0.3.1            digest_0.6.8
## [13] evaluate_0.7         fail_1.2             foreach_1.4.2
## [16] formatR_1.2          genefilter_1.48.1    geneplotter_1.44.0
## [19] grid_3.1.2           gtable_0.1.2         htmltools_0.2.6
## [22] hwriter_1.3.2        iterators_1.0.7      latticeExtra_0.6-26
## [25] MASS_7.3-37          munsell_0.4.2        proto_0.3-10
## [28] RColorBrewer_1.1-2   Rcpp_0.11.6          reshape2_1.4.1
## [31] rmarkdown_0.3.11     RSQLite_1.0.0        scales_0.2.4
```

```
## [34] sendmailR_1.2-1      splines_3.1.2       stringr_0.6.2
## [37] survival_2.37-7      tools_3.1.2         XML_3.98-1.1
## [40] xtable_1.7-4         yaml_2.1.13         zlibbioc_1.12.0
```
